# Mutational analysis of *Aedes aegypti* Dicer 2 provides insights into the biogenesis of antiviral exogenous small interfering RNAs

**DOI:** 10.1371/journal.ppat.1010202

**Published:** 2022-01-06

**Authors:** Rommel J. Gestuveo, Rhys Parry, Laura B. Dickson, Sebastian Lequime, Vattipally B. Sreenu, Matthew J. Arnold, Alexander A. Khromykh, Esther Schnettler, Louis Lambrechts, Margus Varjak, Alain Kohl

**Affiliations:** 1 MRC-University of Glasgow Centre for Virus Research, Glasgow, United Kingdom; 2 Division of Biological Sciences, University of the Philippines Visayas, Miagao, Iloilo, Philippines; 3 School of Chemistry and Molecular Biosciences, The University of Queensland, St. Lucia, Australia; 4 Insect-Virus Interactions Unit, Institut Pasteur, UMR2000, CNRS, Paris, France; 5 Department of Microbiology and Immunology, University of Texas Medical Branch, Galveston, Texas, United States of America; 6 Institute for Human Infection and Immunity, University of Texas Medical Branch, Galveston, Texas, United States of America; 7 Cluster of Microbial Ecology, Groningen Institute for Evolutionary Life Sciences, Groningen, The Netherlands; 8 Australian Infectious Diseases Research Centre, Global Virus Network Centre of Excellence, Brisbane, Queensland, Australia; 9 Bernhard-Nocht-Institute for Tropical Medicine, Hamburg, Germany; 10 German Centre for Infection Research (DZIF), Partner Site Hamburg-Luebeck-Borstel-Riems, Hamburg, Germany; 11 Faculty of Mathematics, Informatics and Natural Sciences, University Hamburg, Hamburg, Germany; 12 Institute of Technology, University of Tartu, Tartu, Estonia; Pennsylvania State University, UNITED STATES

## Abstract

The exogenous small interfering RNA (exo-siRNA) pathway is a key antiviral mechanism in the *Aedes aegypti* mosquito, a widely distributed vector of human-pathogenic arboviruses. This pathway is induced by virus-derived double-stranded RNAs (dsRNA) that are cleaved by the ribonuclease Dicer 2 (Dcr2) into predominantly 21 nucleotide (nt) virus-derived small interfering RNAs (vsiRNAs). These vsiRNAs are used by the effector protein Argonaute 2 within the RNA-induced silencing complex to cleave target viral RNA. Dcr2 contains several domains crucial for its activities, including helicase and RNase III domains. In *Drosophila melanogaster* Dcr2, the helicase domain has been associated with binding to dsRNA with blunt-ended termini and a processive siRNA production mechanism, while the platform-PAZ domains bind dsRNA with 3’ overhangs and subsequent distributive siRNA production. Here we analyzed the contributions of the helicase and RNase III domains in *Ae*. *aegypti* Dcr2 to antiviral activity and to the exo-siRNA pathway. Conserved amino acids in the helicase and RNase III domains were identified to investigate Dcr2 antiviral activity in an *Ae*. *aegypti*-derived Dcr2 knockout cell line by reporter assays and infection with mosquito-borne Semliki Forest virus (*Togaviridae*, *Alphavirus*). Functionally relevant amino acids were found to be conserved in haplotype *Dcr2* sequences from field-derived *Ae*. *aegypti* across different continents. The helicase and RNase III domains were critical for silencing activity and 21 nt vsiRNA production, with RNase III domain activity alone determined to be insufficient for antiviral activity. Analysis of 21 nt vsiRNA sequences (produced by functional Dcr2) to assess the distribution and phasing along the viral genome revealed diverse yet highly consistent vsiRNA pools, with predominantly short or long sequence overlaps including 19 nt overlaps (the latter representing most likely true Dcr2 cleavage products). Combined with the importance of the Dcr2 helicase domain, this suggests that the majority of 21 nt vsiRNAs originate by processive cleavage. This study sheds new light on *Ae*. *aegypti* Dcr2 functions and properties in this important arbovirus vector species.

## Author summary

*Aedes aegypti* mosquitoes that transmit human-pathogenic viruses rely on the exogenous small interfering RNA (exo-siRNA) pathway as part of antiviral responses. This pathway is triggered by virus-derived double-stranded RNA (dsRNA) produced during viral replication that is then cleaved by Dicer 2 (Dcr2) into virus-derived small interfering RNAs (vsiRNAs). These vsiRNAs target viral RNA, leading to suppression of viral replication. The importance of Dcr2 in this pathway has been intensely studied in the *Drosophila melanogaster* model but is largely lacking in mosquitoes. Here, we have identified conserved and functionally relevant amino acids in the helicase and RNase III domains of *Ae*. *aegypti* Dcr2 that are important in its silencing activity and antiviral responses against Semliki Forest virus (SFV). Small RNA sequencing of SFV-infected mosquito cells with functional or mutated Dcr2 gave new insights into the nature and origin of vsiRNAs. The findings of this study, together with the different molecular tools we have previously developed to investigate the exo-siRNA pathway of mosquito cells, have started to uncover important properties of Dcr2 that could be valuable in understanding mosquito-arbovirus interactions and potentially in developing or assisting vector control strategies.

## Introduction

The *Aedes aegypti* mosquito is a widely distributed arbovirus vector [1,2]. It transmits important human-pathogenic arboviruses, including dengue (DENV) and Zika (ZIKV) viruses (*Flaviviridae; Flavivirus*), as well as chikungunya virus (CHIKV; *Togaviridae*; *Alphavirus*) [3–7]. Vector control remains an important tool to combat arboviruses, especially in the absence of vaccines and antivirals for many of these pathogens [8]. As arboviruses actively replicate in their vectors, understanding arbovirus-vector interactions leading to virus infection is essential for vector-based control strategies that impact virus transmission, including targeting vector immune responses that control virus replication [9,10]. Studies on antiviral responses based on RNA interference (RNAi) in insects, such as the critically important exogenous small interfering RNA (exo-siRNA) pathway, were pioneered in *Drosophila melanogaster* [11–16]. This pathway is initiated by sensing and cleavage of viral double-stranded RNA (dsRNA) by the ribonuclease Dicer 2 (Dcr2) that produces mostly 21 nucleotide (nt) virus-derived small interfering RNAs (vsiRNAs). These vsiRNAs are loaded into Argonaute 2 (Ago2), which as part of the RNA induced silencing complex (RISC), retains one vsiRNA strand that targets viral RNA for cleavage. The antiviral and target sensing, as well as cleavage roles of Dcr2 in this pathway, have been established in several key studies [17–24]. In mosquitoes, the exo-siRNA pathway plays an equally important role in controlling arbovirus replication with key characteristics such as viral dsRNA recognition and production of 21 nt vsiRNAs by Dcr2, and an important effector role for Ago2 conserved [25–28]. A possible exception might be ZIKV, where only Dcr2 activity would appear central to controlling virus replication [29–31]. The importance of Dcr2 in mosquito antiviral responses against flaviviruses is also highlighted by the fact that subgenomic flavivirus RNA (sfRNA), a highly structured RNA derived from the 3’ end of the viral genome, inhibits RNAi activities by acting as an RNAi suppressor [32,33]. Other virus-derived small RNAs (vsRNAs) are also generated, including the Dcr2-independent virus-derived PIWI-interacting RNAs (vpiRNAs; 24–30 nt) [34], however their antiviral role is less clear.

**Fig 1:**
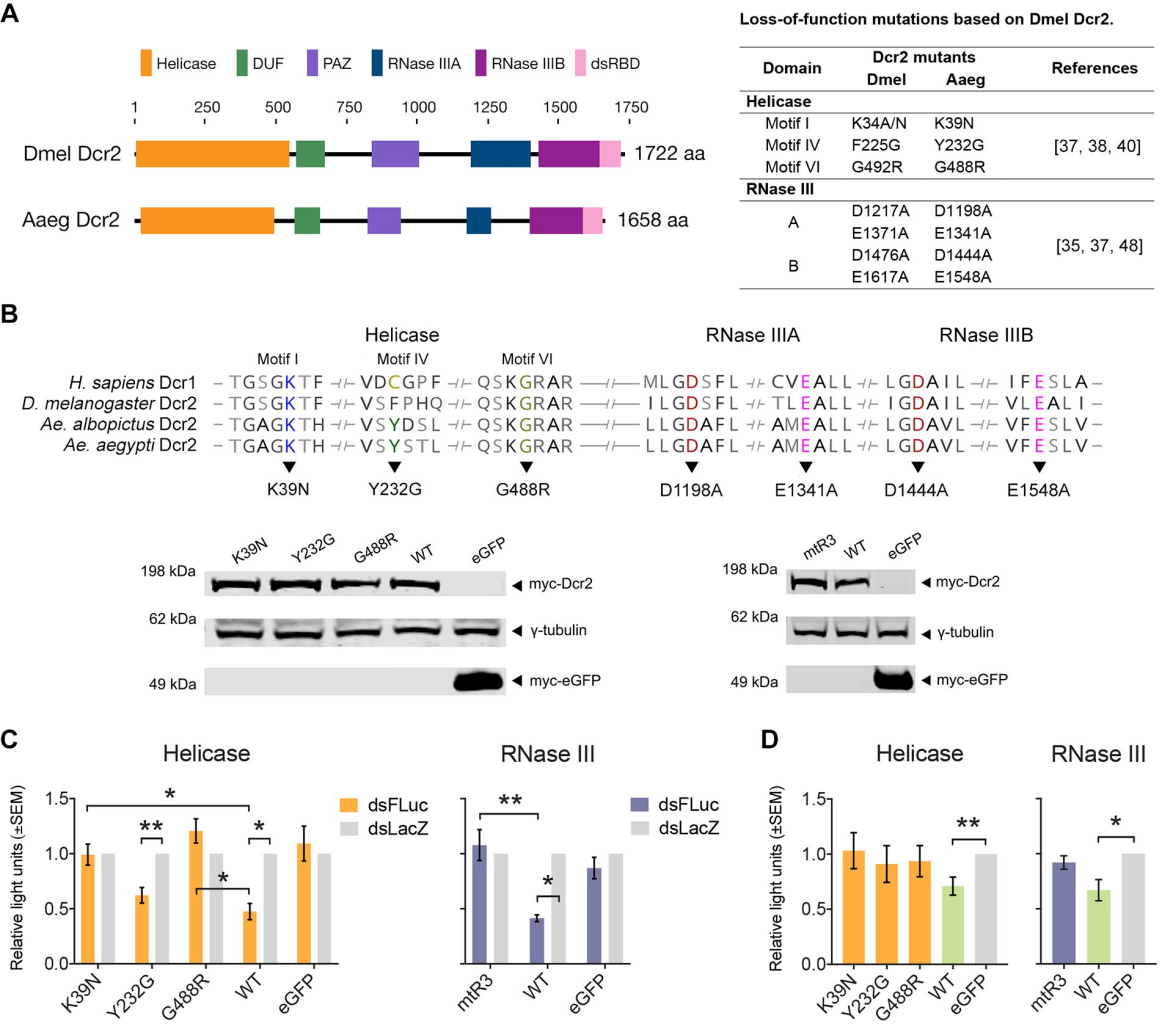
Loss-of-function mutations in Aaeg Dcr2 domains affect the exo-siRNA pathway and antiviral activity. (A) Left panel, schematic diagram of Dmel and Aaeg Dcr2 showing functional domains. Right panel, table showing loss-of-function mutations performed on Aaeg Dcr2 helicase and RNase III domains based on Dmel Dcr2. Abbreviations: DUF = domain of unknown function; PAZ = PIWI-Argonaute-Zwille domain, and dsRBD = dsRNA binding domain. (B) Top panel, multiple sequence alignment of human Dcr1 and insect Dcr2 showing conserved motifs/residues across species with mutagenesis performed on the helicase domain Motif I, IV, and VI that map to Aaeg Dcr2 at K39N, Y232G, G488R, respectively, and the RNase III domain mutations mapping to D1198A, E1341A, D1444A, and E1548A (collectively, mtR3). Bottom panel, immunoblot showing expression of myc-tagged Dcr2 in AF319 cells transfected with pPUb plasmids encoding WT or mutant Dcr2 with pPUb-myc-eGFP (peGFP) as control using anti-myc antibody with γ-tubulin as loading control. Representative blot of n = 2 independent repeats is shown. (C) Silencing activity of mutant Dcr2 assessed by RNAi reporter assay using AF319 cells co-transfected with mutant or WT Dcr2 plasmid (peGFP as control) and FFLuc and RLuc reporter plasmids with dsRNA targeting FFLuc (dsFLuc) or dsLacZ (non-targeting control). At 24 hpt, luciferase levels measured and presented as mean±SEM relative light units (FFLuc/RLuc) versus dsLacZ set to 1 from n = 3 independent repeats with * = p<0.05, ** = p<0.01 versus controls according to two-way ANOVA. (D) Antiviral activity of mutant Dcr2 in AF319 cells (peGFP as control). Cells were infected at 24 hpt with SFV-FFLuc (MOI = 0.1) for 48 h. Measured FFLuc readings shown as mean±SEM relative light units compared to eGFP control set to 1 from n = 3 independent repeats with * = p<0.05, ** = p<0.01 according to Student’s t-test.

Despite the prominent role of Dcr2 in mosquito antiviral defenses, functional data on this effector protein remain very limited. Sequence analysis shows that *Ae*. *aegypti* (Aaeg) Dcr2 is similar in size and shares the same domain organization with *D*. *melanogaster* (Dmel) Dcr2 (Fig 1A): an N-terminal helicase domain followed by a domain of unknown function (DUF), PIWI-Aubergine-Zwille (PAZ) domain, two RNase III domains, and a C-terminal dsRNA binding domain (dsRBD). The helicase domain is necessary for the binding of blunt-ended dsRNA [35]. Moreover, blunt-end dsRNA binding and processing is optimal in the presence of ATP but it is not required for cleavage of dsRNA with 3’ overhangs. Dmel Dcr2-mediated dsRNA cleavage was shown to be supported by its interaction with Loquacious-PD [36]. In addition, cleavage of resistant dsRNAs with structured ends was found to be modulated by Loquacious-PD, allowing cleavage irrespective of dsRNA termini; however, it was not essential for antiviral responses in *D*. *melanogaster* [22,36–38]. Importantly, recognition of termini was crucial to the biogenesis of siRNAs: (1) binding blunt-ended dsRNA through the helicase domain promoting processive siRNA production, or (2) binding of 3’ overhanging dsRNA with the Platform-PAZ domain promoting distributive siRNA production, at least *in vitro* [35,37,38]. The dsRBD was also shown to be required for efficient siRNA production and loading into Ago2 [39].

Recent work has shown interesting differences between Dmel Dcr2 mutations under experimental conditions, with two helicase mutations that abrogated blunt end dsRNA binding *in vitro*. One such mutation (F225G) still allowed the production of vsiRNAs *in vivo* [40], despite the described requirement of the helicase domain in antiviral responses [22,40,41]. This suggests that the cellular environment may contribute to Dmel Dcr2 activity and specificity of the recognition and cleavage processes. In contrast, there is less information about antiviral functions of Aaeg Dcr2 domains, though mutations leading to inactivation have been described in cell lines from a related species *Ae*. *albopictus*, C6/36 and C7/10 [42,43]. These mutations naturally arose in these cell lines, leading to deficiencies in vsiRNA production in response to virus infection [44,45].

Here we took advantage of a recently developed *Ae*. *aegypti*-derived Dcr2 knockout cell line, AF319, in which transient expression of Dcr2 leads to the re-establishment of the silencing phenotype [46]. This directly allows the functional study of the helicase and RNase III domains in this host background and their contributions to antiviral activity and viral dsRNA processing in the exo-siRNA pathway. By identifying conserved amino acids critical for Dmel Dcr2 function and mapping those to Aaeg Dcr2, we identified important residues in the helicase and RNase III domains that are necessary for overall activity in RNAi reporter assays, antiviral function, and vsiRNA production following infection by Semliki Forest virus (SFV; *Togaviridae*, *Alphavirus*), a positive-strand RNA arbovirus. Natural polymorphisms in the helicase and RNase III domain identified from *Dcr2* haplotypes of Asian, African, and American *Ae*. *aegypti* were also assessed and had comparable silencing and antiviral activity. Importantly, functionally relevant amino acids were observed to be conserved throughout mosquito populations and mapped onto a Aaeg Dcr2 structure predicted by AlphaFold. Our data show a vital role for both the helicase and RNase III domains in Aaeg Dcr2 activity wherein: 1) loss of both domain activities strongly affected vsiRNA production and antiviral activity, and 2) loss of helicase function showed that RNase III activity and cleavage of viral dsRNA alone was insufficient to inhibit SFV replication. This demonstrates that the complete exo-siRNA pathway is necessary for antiviral activity against SFV. Specifically, the Aaeg Dcr2 Y232G mutation, equivalent to the Dmel Dcr2 F225G mutation described earlier, retained silencing functionality in cells, although only in an RNAi reporter assay. Loss of Aaeg Dcr2 helicase and RNase III activity resulted in 21 nt small RNAs with piRNA signatures, as opposed to “true”, canonical 21 nt vsiRNAs produced by functional Dcr2. Examination of true 21 nt vsiRNA sequence distribution mapped along the SFV genome and anti-genome, combined with the requirement of helicase functionality for efficient vsiRNA production, suggested that the majority of vsiRNAs are produced by processive cleavage of viral dsRNA by Aaeg Dcr2 in a helicase-dependent manner. This is consistent with the proposed model in *D*. *melanogaster* [38]. Analysis of true 21 nt vsiRNA overlap patterns for functional Aaeg Dcr2 indicated a complex picture, with vsiRNAs frequently overlapping by short (5–7 nt) or long (15–18 nt) stretches, with 19 nt overlaps likely derived from original vsiRNA duplexes generated by Aaeg Dcr2 cleavage also being observed. This reflects a diverse (but consistent) pool of vsiRNAs, which may be critical for the efficiency of antiviral responses.

## Results

### Identification of conserved and functional amino acids in Aaeg Dcr2

Dcr2 proteins are closely related to share functional domains across invertebrate animal species. To determine functionally relevant amino acids in the helicase and RNase III domains, we compared Aaeg Dcr2 to Dmel Dcr2. Multiple sequence alignment of orthologous Dcr2 sequences led to the identification of residues in the helicase domain with K39, Y232, and G488 in Aaeg Dcr2, whose equivalents are critical in Dmel Dcr2 function [35,37,38,40]. Moreover, these residues are part of known helicase motifs [47] that are conserved across divergent species. In addition, D1198, E1341, D1444, and E1548 in Aaeg Dcr2 RNase III A and B domains are well-conserved residues across species that are likely equivalent and have been shown to be functionally important for RNAi activity in Dmel Dcr1 and Dcr2 [37,38,48] (Fig 1A and 1B).

Taking these conserved amino acids, mutations were introduced to Aaeg Dcr2 by mutagenesis cloning of a previously developed expression plasmid, pPUb-myc-Dcr2, encoding a myc-tagged wild-type (WT) Dcr2 (GenBank ID: AAW48725) under the control of a polyubiquitin (PUb) promoter [46] to assess loss of function (Fig 1B, top panel). Mutations individually introduced into the helicase domain were K39N in Motif I, Y232G in Motif IV, and G488R in Motif VI. For the RNase III domains, an expression plasmid with mutations D1198A and E1341A in the RNase IIIA, and D1444A, E1548A in the RNase IIIB domain were collectively introduced and called mutant RNase III (mtR3), with mutations to alanine expected to inactivate RNase III activity. Sorting Intolerant From Tolerant (SIFT) algorithm [49] prediction scores suggested that the individual mutations would affect Dcr2 function (S1 Table). The expression of Dcr2 was verified by immunoblotting lysates obtained from AF319 Dcr2 knockout cells transfected with mutant or WT Dcr2 expression plasmids, with pPUb-myc-eGFP (peGFP) as control (Fig 1B, bottom panel).

To determine the effect of the mutations on Dcr2 silencing activity, a plasmid-based RNAi reporter assay was performed [46]. Briefly, AF319 cells were co-transfected with firefly luciferase (FFLuc) reporter (and *Renilla* luciferase, RLuc, expressing control plasmid) and WT or mutant Dcr2 plasmids (with peGFP as control) together with dsRNA targeting FFLuc (dsFLuc) or a non-silencing control (dsLacZ). At 24 h post-transfection (hpt), it was observed that all mutants except Y232G mediated no silencing activity against FFLuc and were comparable to dsLacZ-transfected controls (Fig 1C). Specifically, Y232G retained residual silencing that was comparable to WT Dcr2. Its equivalent mutant in Dmel Dcr2 (F225G) also induced vsiRNA production *in vivo* [40]. The findings suggested that the residues previously identified from studies on Dmel Dcr2 were also important in Aaeg Dcr2 activity, and both helicase and RNase III domain activity appeared to be critical in the overall silencing function and exogenous dsRNA cleavage of Aaeg Dcr2. To verify the functionality of the exo-siRNA pathway downstream of mutant Dcr2, mutations K39N and mtR3 were assessed following transfection of siRNA targeting FFluc in a plasmid-based reporter assay (S1 Fig). Silencing activity was readily detected compared to the control, suggesting that processes post dsRNA cleavage and siRNA loading into Ago2 remained active.

The ability of the loss-of-function mutants to limit arbovirus replication was also examined. The replication of SFV-FFLuc (MOI = 0.1) reporter virus in AF319 cells transiently expressing mutant or WT Dcr2 was assessed (Fig 1D). At 48 h post-infection (hpi), expression of WT Dcr2 resulted in significant reduction of SFV replication compared to eGFP-transfected control cells; no mutant Dcr2 was able to reduce SFV replication as determined by FFLuc levels relative to the eGFP-transfected controls. Including Y232G, which showed some activity in the RNAi reporter assay as mentioned above.

### Naturally occurring *Dcr2* polymorphisms in field-derived *Ae*. *aegypti* mosquitoes

**Fig 2:**
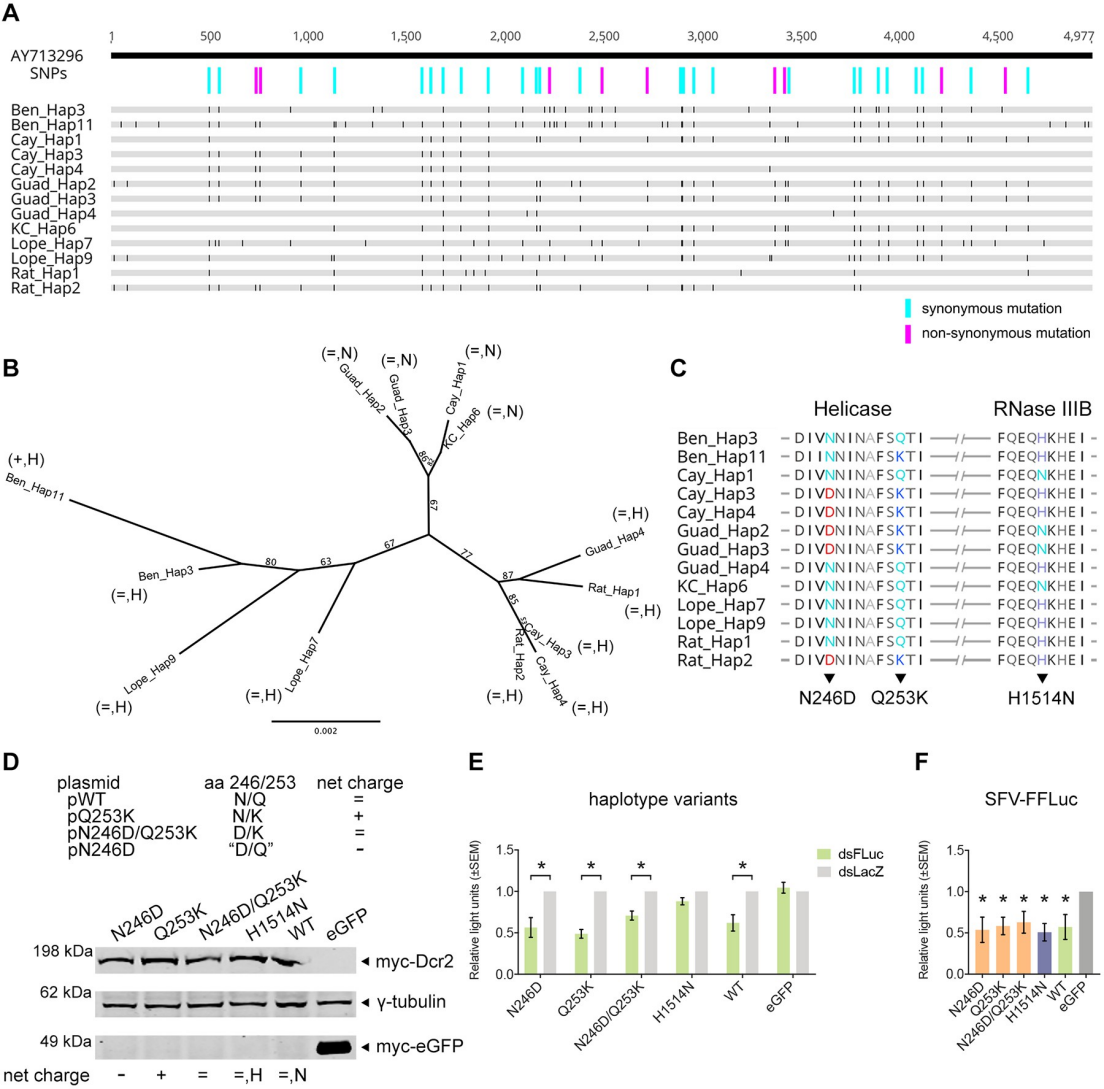
Naturally occurring *Dcr2* variants among field-derived mosquito populations. (A) *Dcr2* haplotypes (in grey) were identified from early generation colonies of *Ae*. *aegypti* initially collected from Cameroon (Ben), French Guiana (Cay), Guadeloupe (Guad), Cambodia (KC and Rat), and Gabon (Lope) and aligned to a reference WT *Dcr2* sequence (GenBank ID: AY713296; thick black line) to determine SNPs. Black bars show disagreements to WT *Dcr2* with silent and non-synonymous SNPs highlighted by cyan and magenta bars, respectively. (B) Phylogenetic tree of putative amino acid sequences of haplotypes highlighting the amino acid net charge at positions 246/253 (+: positive, -: negative, =: neutral) and an H or N at position 1514. Numbers on branches indicate % consensus support with the substitution rate measured by the scale bar. (C) Alignment of haplotypes highlighting amino acid variants N246D and Q253K in the helicase, and H1514N in the RNase IIIB domain. (D) Mutagenesis of WT Dcr2 to generate amino acid mutations at position 246/253 (aa 246/253) and a theoretical "D/Q" variant (N246D; negative charge) not observed among the surveyed mosquito populations. To check for Dcr2 expression, AF319 cells transfected with variant or WT Dcr2 (peGFP as control) were lysed and probed for myc-Dcr2 by immunoblot using anti-myc tag and γ-tubulin (loading control) antibodies. Representative blot of n = 2 independent repeats is shown. (E) RNAi reporter assay was performed to determine the effect of the variants on silencing activity. AF319 cells co-transfected with WT or variant Dcr2 plasmids (peGFP as control) and FFLuc and RLuc reporter plasmids with dsRNA targeting FFLuc (dsFLuc) or dsLacZ (non-targeting control). At 24 hpt, luciferase levels were measured and shown as mean±SEM relative light units (FFLuc/RLuc) versus dsLacZ set to 1 compared to eGFP control from n = 3 independent repeats with * = p<0.05 according to two-way ANOVA. (F) Antiviral activity of Dcr2 variants against SFV-FFLuc (MOI = 0.1) was determined in AF319 cells transiently expressing variant or WT Dcr2 (peGFP as control). Cells were infected 24 hpt. At 48 hpi, FFLuc readings were measured and presented as mean±SEM relative light units compared to eGFP control set to 1 from n = 3 independent repeats with * = p<0.05 versus control according to two-way Student’s t-test.

To determine the degree of conservation of the helicase and RNase III domains, *Dcr2* cDNA from adult female *Ae*. *aegypti* originating from Cameroon (Ben), French Guiana (Cay), Guadeloupe (Guad), Cambodia (KC and Rat), and Gabon (Lope) were sequenced. Thirteen haplotype sequences were identified (S2 Table) and examined further for single nucleotide polymorphisms (SNPs) by comparing them to our WT *Dcr2* reference sequence (GenBank ID: AY713296) that was previously cloned to obtain pPUb-myc-Dcr2 [46]. SNP analysis showed that the majority of the polymorphisms were silent transition changes, but it was observed that all field-derived mosquito *Dcr2* had a C variant nucleotide at position 1684 (S3 Table). In addition, SNPs were identified spanning the entire length of *Dcr2* with a number resulting in amino acid substitutions (Fig 2A).

A phylogenetic tree of the putative amino acid sequences of the *Dcr2* haplotypes showed a higher degree of genetic diversity between Aaeg Dcr2 of African origin compared to those of Asian or American origin (Fig 2B), although pairwise identity between haplotype genetic sequences was fairly high (>99%; S2 Fig). Three major clusters were observed, with all representative haplotypes from Cameroon and Gabon mosquito populations belonging to one clade. Interestingly, Dcr2 amino acid mutations at positions 246 and 253 appear to occur in pairs, with either an N246/Q253 or D246/K253 (Fig 2C). These combinations result in a neutral amino acid net charge except for Ben_Hap11 (N246/K253), resulting in a positive net charge. Another distinct polymorphism was in the RNase IIIB domain at position 1514 where either an H or N was observed. Other polymorphisms were mainly in regions between domains. Importantly, amino acids identified above as critical for helicase and RNase III activities were conserved across *Ae*. *aegypti* populations.

Mutations based on the observed net charge conservation at amino acid 246 and 253 were introduced to pPUb-myc-Dcr2 with the addition of a theoretical negative charge mutant (N246D) not observed among the mosquito populations surveyed (Fig 2D, top panel). Site-directed mutagenesis cloning was performed, and the expression of the myc-tagged variants in AF319 cells was verified by immunoblotting (Fig 2D, bottom panel). The predicted effect of the natural haplotype variants on protein function was also assessed by SIFT, with scores indicating tolerated substitutions (S1 Table). To test the silencing efficiency of these natural haplotype variants, the same RNAi reporter assay mentioned above was performed. The assay revealed that all except H1514N could silence FFLuc expression and were comparable to the WT (Fig 2E). The variants were also investigated for their antiviral capacity against SFV. Transient expression of the natural haplotype variants in AF319 significantly reduced SFV-FFLuc (MOI = 0.1) levels at 48 hpi versus eGFP-expressing control cells, with the antiviral activity of the variants not significantly different from WT Dcr2 (Fig 2F). Interestingly, the non-silencing H1514N variant could limit SFV replication, which could be related to how Aaeg Dcr2 processes transfected dsRNA versus viral dsRNA. Although both are considered exogenous dsRNA, it has been shown that Dmel Dcr2 performs termini selective cleavage [37,38]. Overall, these findings indicate that the functionality of the helicase and RNase III domains is important, and thus conserved.

### The putative structure of *Ae*. *aegypti* Dcr2

**Fig 3:**
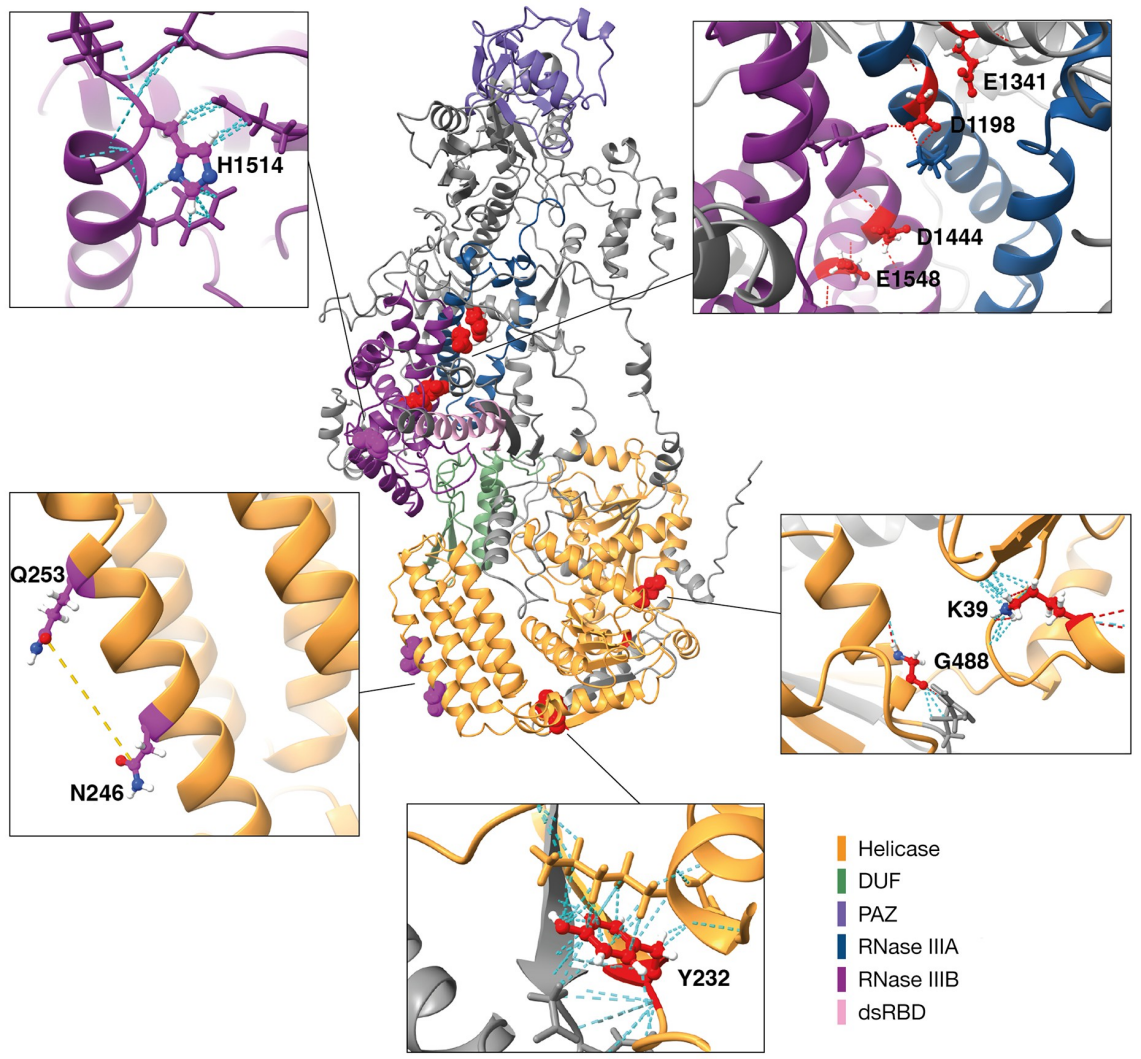
Locations of conserved amino acids critical for Aaeg Dcr2 function can be predicted and are distributed between surface and buried residues. Center, AlphaFold prediction (mean pLDDT score = 81.4) for the structure of WT Aaeg Dcr2 (GenBank ID: AAW48725), colored by predicted domains from annotations and Interproscan. Residues representing conserved amino acids involved in Dcr2 activity and natural haplotype variants are highlighted in red and purple, respectively. Inset panels show enlarged representations of conserved amino acid residues targeted in mutagenesis experiments, with dashed lines showing contacts in red, hydrogen bonds in cyan, and distances in yellow. Clockwise from upper right: loss-of-function mutations in the RNase III domains line a predicted cleft in the core of the protein; G488 and K39 are forecast to be buried near the surface of the helicase domain; Y232 appears to form hydrogen bonds and extensive contacts with a neighbouring α-helix (residues 360–374) and β-strand (residues 496–502); natural haplotype variant residues N246 and Q253 (distance = 10.71Å) are likely positioned on the exposed surface of the helicase domain; H1514 is seen at the surface exposed tip of an α-helix.

The structure of Aaeg Dcr2 was predicted, in order to locate the conserved amino acids important for Dcr2 activity (Fig 3 and S1 Movie). Results of AlphaFold inferencing produced a model with a mean predicted local distance difference test (pLDDT) value of 81.4 (S3 Fig). This indicates a confident prediction, according to guidelines set out on EMBL’s AlphaFold Protein Structure Database [50], available at https://alphafold.ebi.ac.uk. By examining the structure and assigning domain predictions (based on GenBank ID: AAW48725 annotations and InterProScan in Geneious Prime), it can be seen that the putative structure contains two main globular modules: a “foot” module comprising of the predicted helicase domain (yellow) and a “body” domain comprising of the two forecasted RNase IIIA (blue) and IIIB (purple) domains, and the PAZ (violet) domain protruding at the far end. The two domains are bridged by a predicted domain of unknown function (DUF; green). The interface between the two modules forms a cleft, partially occluded by the projected dsRNA binding domain (dsRBD; pink). A previous study [38] yielded cryo-EM maps of moderate resolution of Dmel Dcr2. Fitting the apo-Dcr2 map (EMDB entry 7291) from this publication to our model (S3C Fig; ~50% of atoms inside contour at contour level 0.0066) illustrated that the overall architecture of Dmel Dcr2 and our model is shared (S3D Fig). The location of several conserved amino acids, for example, those in the helicase domain, on the Dcr2 surface suggests that they could be involved in interactions with other proteins, though this remains to be investigated. For completeness, the locations of haplotype variants were also located in the predicted Aaeg Dcr2 structure (Fig 3).

### Role of Dcr2 helicase and RNase III domains in viral dsRNA targeting and vsiRNA production

**Fig 4:**
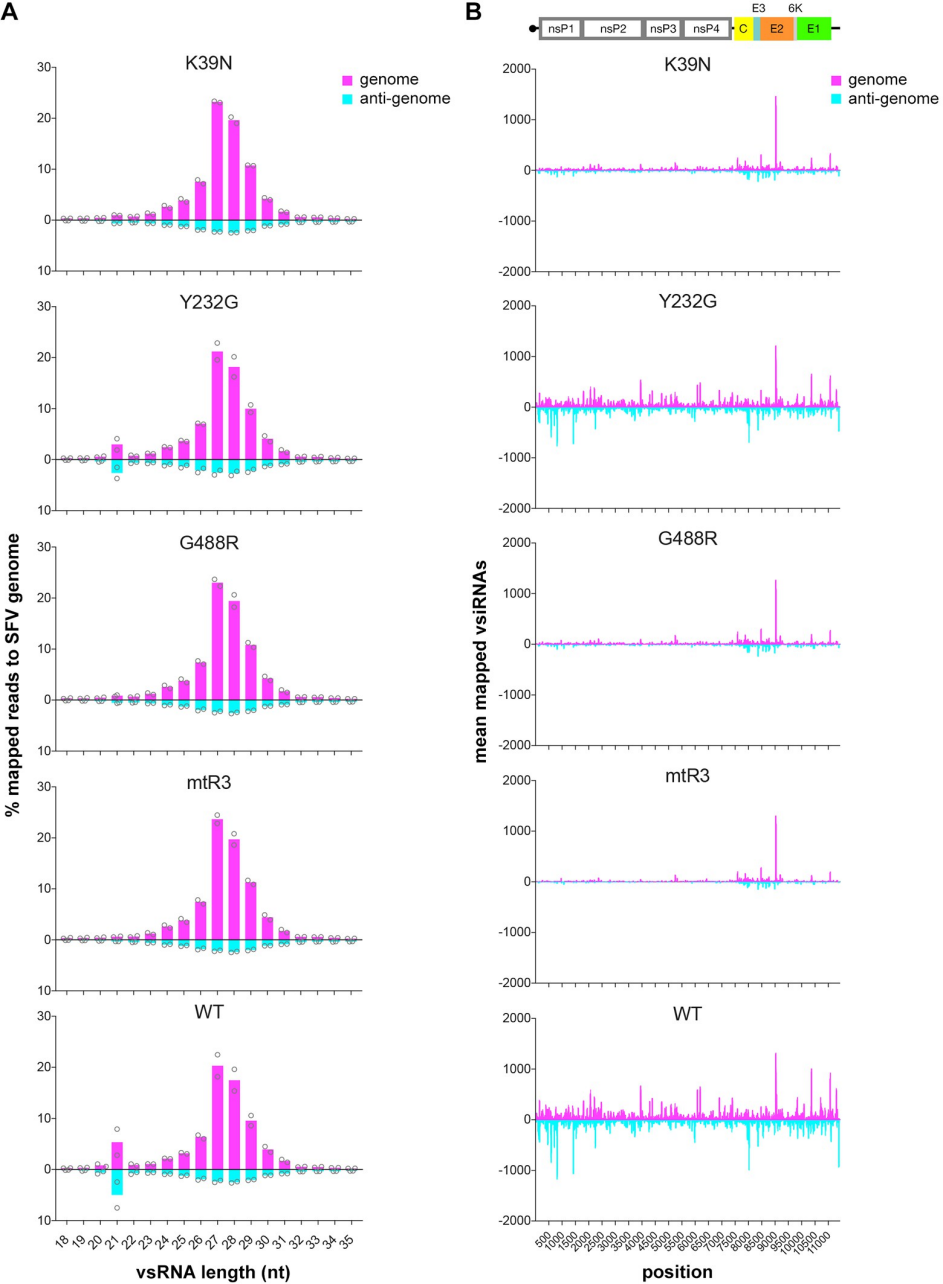
Domain loss-of-function mutations in Aaeg Dcr2 affect vsiRNA production. (A) Distribution of 18–35 nt vsRNAs mapped to the SFV (GenBank ID: KP699763) genome (sense; magenta) or anti-genome (anti-sense; cyan) shown as mean % mapped reads in bars with replicates shown as circles from n = 2 independent repeats from AF319 cells transiently expressing mutant or WT Dcr2 and infected with SFV (MOI = 1) at 48 hpi. (B) SFV-derived 21 nt vsiRNAs from (A), mapped to SFV genome (magenta) or anti-genome (cyan) with the mean number of mapped reads shown from n = 2 independent repeats. The SFV genome organization (top panel) is shown for reference.

Next, we assessed whether the loss of silencing ability of the helicase or RNase III mutants were linked to changes in vsiRNA production. In mosquito cells, the Dcr2-dependent exo-siRNA response to arbovirus infection results in the production of predominantly 21 nt vsiRNAs that map to the viral genome and anti-genome [26–28]. Similar to our previous data [46], expression of WT Dcr2 in AF319 cells followed by SFV infection resulted in the production of 21 nt vsiRNAs that mapped to the SFV genome and anti-genome (Fig 4A). An obvious difference between samples from cells with WT and mutant Dcr2 was the depletion of 21 nt vsiRNAs that could be mapped to the SFV genome and anti-genome (Fig 4B). Mutations K39N and G488R in the helicase domain and mtR3 in the RNase IIIA and B domains resulted in a substantial decrease of SFV-derived vsiRNAs. In contrast, mutant Y232G showed better production of vsiRNAs compared to the other mutants but fewer in number than the WT. This may explain the silencing ability observed in the RNAi reporter assay but is insufficient for the silencing of SFV replication in our experimental set-up.

Generally, vsRNAs in the canonical vpiRNAs size range (24–30 nt) mapping to the SFV genome were abundant, as we previously observed [46], though their contribution to controlling virus replication remains unclear. These exo-siRNA pathway-independent vpiRNAs are linked to the piRNA pathway. Our vsRNA data also detected vpiRNAs that were abundant regardless of Dcr2 sequence and functionality (S4A Fig). Importantly, vpiRNAs display a nucleotide bias of an A in position 10 on the genome strand and U in position 1 of the anti-genome strand (A_10_/U_1_); with a 10 nt overlap between genome and anti-genome strands, which is due to the ping-pong amplification mechanism of the piRNA pathway [34]. Indeed, this characteristic signature was detected here in 27 nt vpiRNAs (representative for vpiRNAs; S4B Fig). Summaries of numbers and percentages of all small RNAs mapped to SFV are shown in the S4 Table.

### Characterisation of 21 nt vsiRNAs with piRNA signature: vsi-piRNAs

**Fig 5:**
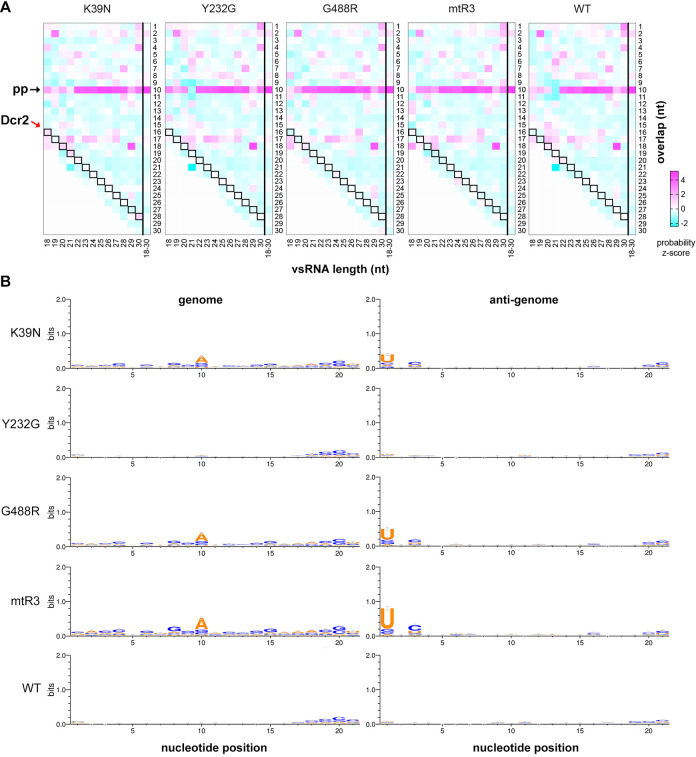
Characterising 21 nt vsiRNAs with vpiRNA signature: vsi-piRNAs. (A) Heat maps showing mean overlap probabilities of z-scores of 18–30 nt SFV-derived vsRNAs from AF319 transiently expressing mutant or WT Dcr2 from n = 2 independent repeats. vsRNA lengths are shown horizontally, and the number of nucleotide overlaps listed vertically. Red arrow labelled Dcr2 indicates expected 2 nt overlap from dsRNA cleavage with cells boxed in black. Black arrow labeled pp (ping-pong) shows expected 10 nt overlap from potential ping-pong amplification. (B) Representative sequence logos of n = 2 independent repeats (other replicate in S5 Fig) of 21 nt vsRNAs mapping to the genome (left panel) or anti-genome (right panel) of SFV from AF319 transiently expressing mutant or WT Dcr2. Overall height of the stack indicates conservation presented as bits, with the height of each symbol reflecting the relative frequency of the corresponding nucleotide at a given position. Error bars indicate an approximate Bayesian 95% credible interval.

Mutations in the helicase and RNase III domains clearly depleted vsiRNA production, with only a few 21 nt reads still detectable, predominantly in the region matching the start of the subgenomic RNA. To investigate the origins of these 21 nt vsiRNAs, their sequences were characterized based on overlap probabilities using a previously developed algorithm that maps vsRNA duplexes by generating overlapping pairs between sequences [51]. This analysis revealed a strong bias towards a 10 nt overlap for 21 nt vsiRNAs produced in cells transfected with mutant Dcr2, except in the Y232G mutant and WT Dcr2 (Fig 5A). This 10 nt bias is reminiscent of the Dcr2-independent ping-pong amplification observed in piRNAs [34,51], which is apparent in the longer vsRNAs (24–30 nt). This suggested a ping-pong biogenesis of these 21 nt vsiRNAs detected in cells with a dysfunctional Dcr2. Sequence analysis of these 21 nt vsiRNAs also showed the classic A_10_/U_1_ bias for all Aaeg Dcr2 mutants except Y232G (Figs 5B and S5) that was also observed in the 27 nt vpiRNAs (S4B Fig) mapping to the viral genome and anti-genome. These 21 nt vsiRNAs with piRNA signature are not true vsiRNAs and will be referred to as “vsi-piRNAs”, separating the 21 nt vsRNAs into two groups. Such 21 nt vsi-piRNAs may be produced in presence of WT Dcr2 or mutant Y232G (which functions similarly to WT in terms of dsRNA cleavage but less efficiently). Still, the signature signal is likely outnumbered by “true”, canonical 21 nt vsiRNAs.

Previous work in Aag2 cells had led to the identification of *Ae*. *aegypti* orthologs of the endonuclease Zucchini and exonuclease Nibbler, both involved in piRNA 3’ end formation in *D*. *melanogaster*. Zucchini-mediated piRNA 3’ end formation depends on sequence motif recognition and preferably a U downstream of the cleavage site. This results in piRNA sharp ends and a so-called +1U bias. Importantly, analysis of vpiRNAs from Aag2 cells infected with the alphavirus Sindbis (SINV) had suggested a Zucchini-like biogenesis mechanism in vpiRNA 3’ termini formation, as sharp ends and +1U bias were observed [52]. This led us to further investigate the origins of 21 nt vsi-piRNAs by subjecting vpiRNAs (25–30 nt) and 21 nt vsi-piRNAs sequences to a similar analysis. Strikingly, following SFV infection of AF319 cells, we observed +1U bias in 25–30 nt vpiRNAs but not across 21 nt vsRNAs (S6 Fig), suggesting common vpiRNA production mechanisms across alphaviruses in *Ae*. *aegypti*-derived cells, regardless of the presence of mutant or WT Dcr2.

### Analysis of true 21 nt vsiRNAs revealed predominant patterns of sequence overlaps

**Fig 6:**
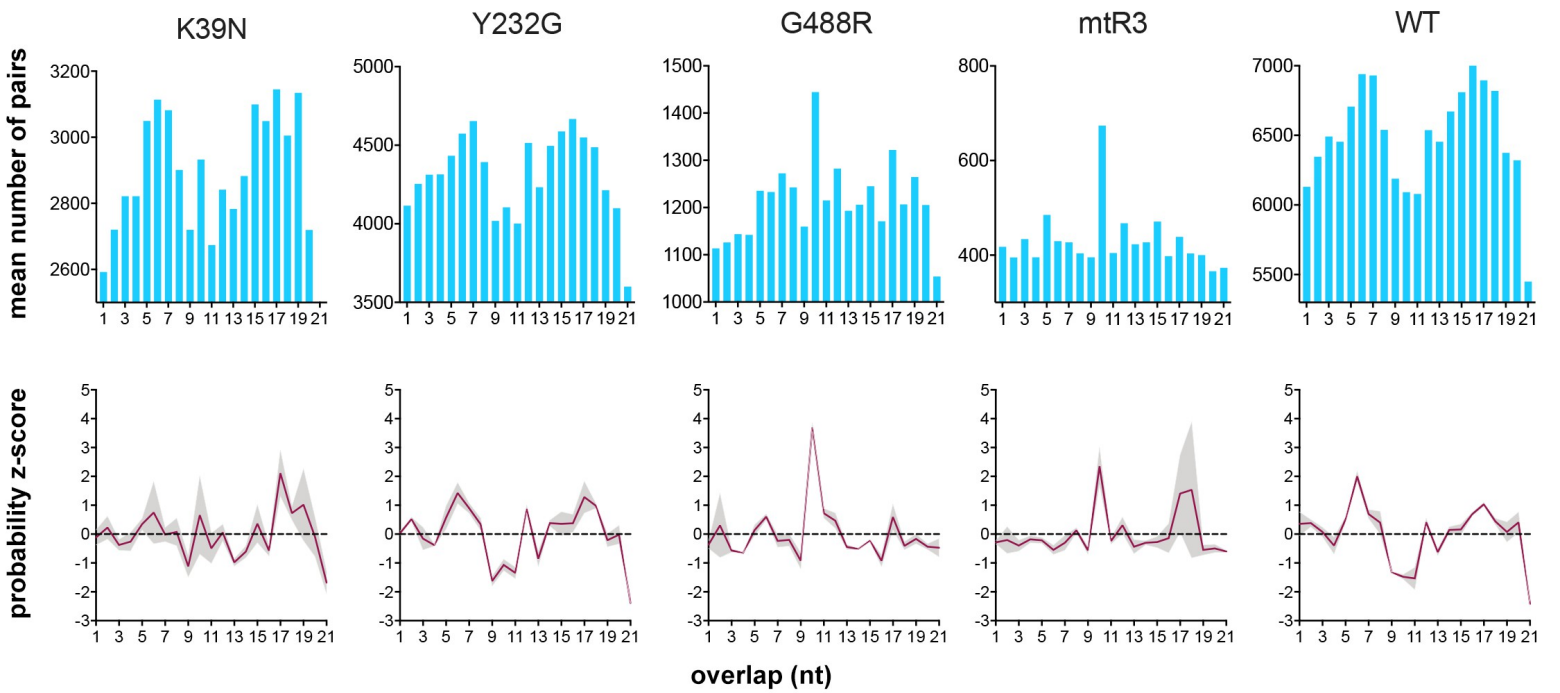
Viral dsRNA cleavage pattern of Aaeg Dcr2 during SFV infection. Number of overlapping pairs (top panel) and probability z-scores (bottom panel) of 21 nt virus-derived small RNAs from AF319 transiently expressing mutant or WT Dcr2 infected with SFV. Mean of n = 2 independent repeats is presented as bars or red line (grey region indicates the range).

Virus-derived dsRNA cleavage by Dmel Dcr2 has been shown to result in predominant vsiRNA sequences with a distinct 19 nt overlap between small RNA duplexes with 2 nt overhangs, presumably vsiRNA duplexes [51,53,54]. Other previous work has since led to a model for Dmel Dcr2 where the helicase domain recognizes blunt end viral dsRNA, and processive dsRNA cleavage leading to diverse vsiRNAs [55]. The distribution of helicase domain-dependent, true 21 nt vsiRNAs produced by WT Aaeg Dcr2 in AF319 cells along the SFV genome and anti-genome also suggests a processive cleavage mechanism covering the entire length of the viral RNA (Fig 4B). By using the overlapping pairs analysis mentioned earlier, the extent of overlap of 21 nt vsiRNA sequences was determined to investigate vsiRNA production patterns and phasing. A pattern was observed in the number of overlapping pairs wherein sequences of true 21 nt vsiRNAs produced by WT Dcr2 predominantly overlapped by either short stretches of 5–7 nt or longer stretches of 15–18 nt as confirmed by the z-scores of the overlaps and the corresponding probability z-scores (Figs 6 and S7A).

This suggests that viral dsRNA cleavage by Aaeg Dcr2 results in diverse vsiRNA sequences with a wide range of overlaps. However, 19 nt overlaps were also detected, which may denote the original vsiRNA duplexes. Whether this finding represents critical differences to observations with Dmel Dcr2 [51,53,54] requires further investigation. In addition, the overlap pattern generated by mutant Y232G showed a striking resemblance to WT Dcr2, corroborating further that this mutant can still produce true 21 nt vsiRNAs. In contrast, for the other mutant Dcr2 we identified more conspicuous patterns with 10 nt overlaps as mentioned earlier, which had low z-score probabilities for 21 nt vsRNAs with 5–7 and 15–18 nt overlap, unlike what was observed for WT and Y232G Dcr2. This further confirms that these mutant Dcr2 had lost their functionality, and presumably, only a few vsiRNAs are produced and masked by vsi-piRNAs. To verify these findings, we re-analyzed previously published small RNA data of SFV infected AF319 cells, AF319 cells transiently expressing WT Dcr2, and the parental AF5 cells that naturally express Dcr2 [46]. The re-analysis gave similar results for true 21 nt vsiRNAs (S7B Fig) following a comparable pattern of overlapping pairs (S7C Fig), suggesting a degree of consistency by which functional Dcr2 processes SFV-derived dsRNA in *Ae*. *aegypti* cells.

### Functional Aaeg Dcr2 generated diverse but consistent true 21 nt vsiRNAs during SFV infection

**Fig 7:**
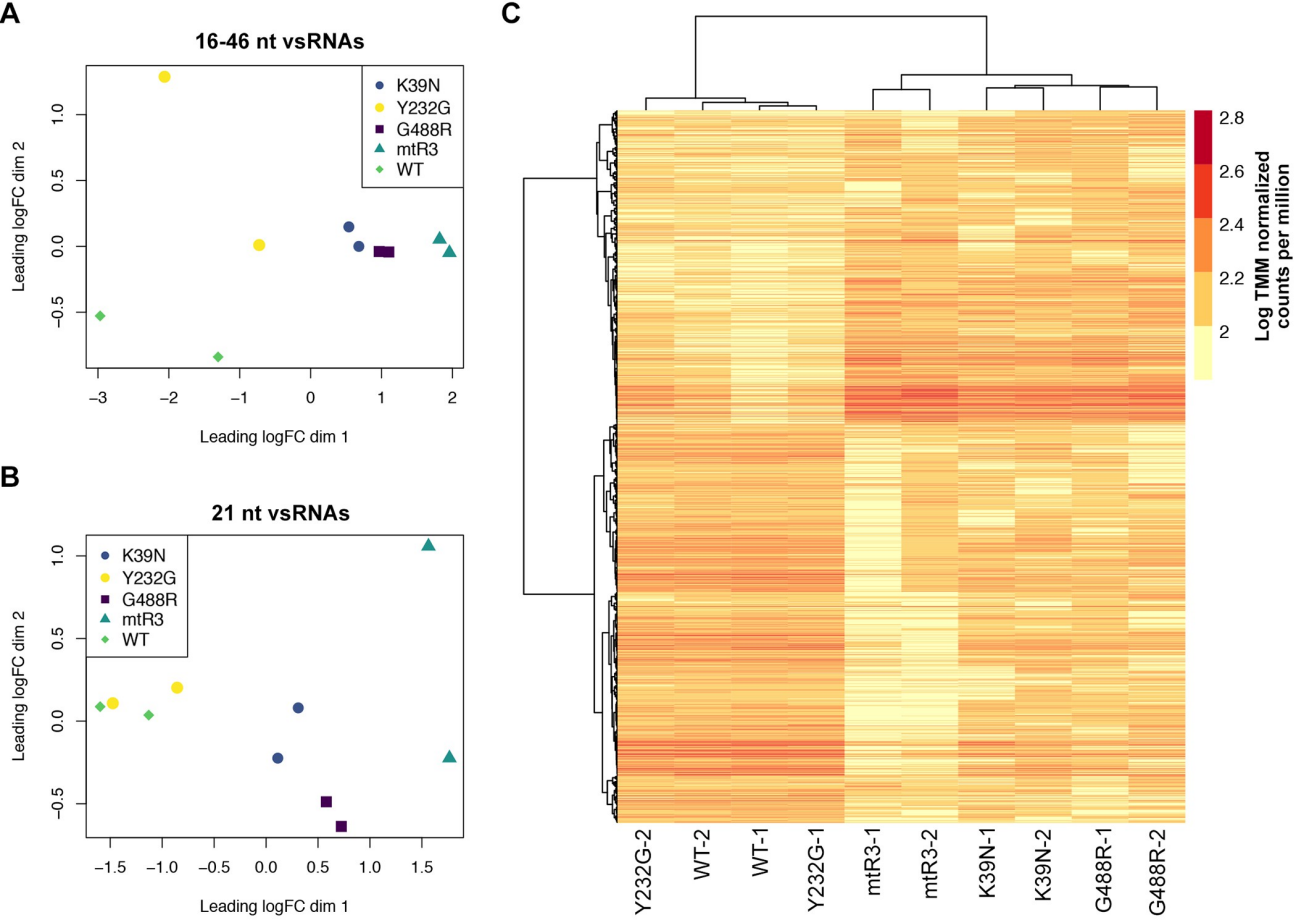
Functional Aaeg Dcr2 generates diverse but highly consistent true 21 nt vsiRNAs. (A) Multi-dimensional scaling (MDS) plot of vsRNAs (16–46 nt) and (B) 21 nt vsRNAs from AF319 cells transiently expressing functional Dcr2 (WT or Y232G; mainly true 21 nt vsiRNAs) or mutant Dcr2 (G488R, K39N, mtR3; mainly vsi-piRNAs) per replicate with axes presented as log fold change (LogFC). (C) Heatmap showing the relationship of 21 nt vsRNAs generated from AF319 cells transiently expressing WT or mutant Dcr2 (as indicated above) during SFV infection. Hierarchical clustering of treatment replicates (columns) and 21 nt vsRNAs (rows) performed by first calculating a distance matrix using Pearson correlation distance and clustered using Ward’s minimum variance method. Each library is shown, and the scale corresponds to log-transformed TMM normalized counts per million.

To explore the possibility that Aaeg Dcr2 loss-of-function mutations affected population-level diversity of SFV vsRNA we conducted a differential transcript abundance analysis using edgeR (Fig 7). To determine this, we treated all the SFV-mapped small RNA reads (16–46 nt) as unique transcripts and generated count tables with normalized reads for all vsRNAs or 21 nt vsRNA subset (S7D Fig). Counts were then normalized for library size using the weighted trimmed mean of M-values (TMM) method and visualized in a multi-dimensional scaling (MDS) plot of the samples. MDS plots can be viewed as a type of unsupervised clustering where distances correspond to average log-fold changes between each pair of samples. Interpretations of the MDS plot indicated that the distances (and therefore similarity) between each sample mutant or WT Dcr2 correlate. This indicated a similar population of vsRNAs (16–46 nt) within treatments, except for SFV derived vsRNAs from cells transiently expressing WT and Y232G Dcr2. Indeed, WT and Y232G showed the most variation in vsRNA populations, suggesting greater diversity of vsRNAs compared to dysfunctional Dcr2 mutants K39N, G488R, and mtR3 (Fig 7A).

When 21 nt vsRNAs were isolated independently, the pattern was comparable for mutant Dcr2. However, true 21 nt vsiRNAs from WT and mutant Y232G were highly similar (Fig 7B) and grouped together. The 4 samples from the WT and Y232G clustered closely across both leading log dimensions. In contrast, the remaining dysfunctional mutants had greater inter-treatment diversity and grouped separately within the MDS space. Importantly it could also be observed that the greatest dissimilarity between replicates of any sample was 21 nt vsRNAs generated by mtR3, as shown by the plot versus K39N and G488R. This inter-treatment grouping is further illustrated in the clustered heatmap (Fig 7C). One striking difference between treatments is that select pools of 21 nt vsRNAs generated by WT and Y232G were predominantly absent in the other mutants. Moreover, there are 21 nt vsRNAs that were generated across treatments but differed greatly in the number of reads. It can also be observed that the two pools of 21 nt vsRNAs were markedly different between functional and dysfunctional Aaeg Dcr2. Overall, this shows that in the presence of functional Aaeg Dcr2 (WT and Y232G), diverse pools of vsRNA populations are produced during SFV infection, though pools of true 21 nt vsiRNAs were highly consistent.

## Discussion

Understanding specific aspects of the antiviral exo-siRNA pathway in *Ae*. *aegypti* has suffered from a lack of molecular tools compared to *D*. *melanogaster*. Here we used our recently developed Dcr2 knockout *Ae*. *aegypti* cell line (AF319 cells) and expression system to understand the functionality and contribution of key domains in the effector protein, Dcr2 [26–28]. While the overall role of mosquito Dcr2 has been extrapolated from Dcr2 orthologs and related Dicer enzymes, few functional studies have been carried out in mosquito systems. Indeed, the absence of 21 nt vsiRNAs in *Ae*. *albopictus* derived cell lines containing deficiencies in Dcr2 has been the main demonstration of mosquito Dcr2 antiviral function comparable to that in *D*. *melanogaster* [42–45]. We developed a study based on the extensively examined Dmel Dcr2 [55], investigating comparative properties between the orthologous proteins. With the recent discovery of Dcr2-interacting proteins in *Ae*. *aegypti* [56], Aaeg Dcr2 may possess a separate dynamic exclusive to this vector species, and we provide tools and baseline data for such studies. The development of AF319 cells, which allows Dcr2 expression and reconstitution of activities [46], has opened new possibilities to examine Dcr2 functionalities and its importance in *Ae*. *aegypti*. We investigated conserved amino acids in the Dcr2 helicase and RNase III domains to assess the targeting of SFV-derived dsRNA and biogenesis of vsiRNAs that were identified to be homologous and functionally important residues to Dmel Dcr2. In addition, critical amino acids were found to be conserved in *Dcr2* haplotype sequences determined from field-derived *Ae*. *aegypti* representative of mosquito populations from Asia, Africa, and the Americas.

Mutations in the Rnase IIIA and IIIB domains (mtR3) largely abrogated 21 nt vsiRNA production as shown by small RNA sequencing, thus confirming its critical role in viral dsRNA cleavage. Equally, helicase-affecting mutations showed that cleavage of dsRNA alone was insufficient for silencing and this activity is required for targeting SFV replication in the exo-siRNA pathway. Specifically, motif I and motif VI of the helicase domain appear to be more vital than motif IV as mutations in the conserved K and G residues, respectively, resulted in both poor dsRNA-triggered silencing and antiviral response. Both motifs are involved in ATP binding and hydrolysis [57]. The role of the helicase domain in Dmel Dcr2 has been analyzed in detail, with specific mutations that abolish functionality and binding of blunt-ended dsRNA characterized [35,37,38]. Moreover, specific polymorphisms we identified in the helicase and Rnase IIIB domain from *Dcr2* haplotype sequences retained their antiviral activity, indicating that the functionality of these Dcr2 domains is important. These data strengthen the argument of the importance of conserved functions of these domains in Aaeg Dcr2.

The AlphaFold based prediction of Aaeg Dcr2 opens new opportunities for understanding targeting, and processing of dsRNA. Two areas of significant difference between Dmel Dcr2 [38] and our model were observed, highlighting possible insights into Dcr2 structure (S3 Fig). First, the region from amino acids 1018–1069 is visualized in our model as a mostly unstructured loop, spanning between the two modules. Taken together with the low pLDDT values from AlphaFold (suggesting low confidence in local predictions for this region) and the absence of density in this region in the EM map, it seems reasonable to suggest that this region is flexible and disordered. Secondly, there is a discrepancy in the “foot” module between our model and the Dmel Dcr2 structure; in this region, both electron density and atomic model are partly unaccounted for. Predicted aligned error (PAE) statistics for our model show high confidence in the relative orientations of residues *within* the “body” and “foot” modules but poor confidence in the relative orientations of the two modules. Alongside electron density data from Dmel Dcr2, this suggests that the “body” and “foot” domains maybe be flexibly permuted with respect to one another. Furthermore, the PAE statistics show a crossover region with moderate to high confidence with respect to both modules. This region almost exactly corresponds to the predicted DUF, bridging between the “body” and “foot” modules. This provides a persuasive explanation for the function of this domain as a hinge or pivot, allowing the relative rearrangement of the two structure modules. The results of this study demonstrate the utility of AlphaFold to generate novel hypotheses, and in this case allowing locations of conserved amino acids to be determined that are important for Aaeg Dcr2 function. This will help in understanding the contributions of conserved residues and motifs to Dcr2 activities.

Previous work has suggested that the helicase-dependent processive cleavage of Dmel Dcr2 could lead to diverse pools of vsiRNAs as dsRNA threads through the helicase domain, producing shorter and heterogenous threading intermediates as by-products affecting the phase of siRNA production [38]. Indeed, as previously suggested, the precise nature of dsRNA termini in viral infections remains to be determined [40], but precise blunt ends would have to be generated during viral genome replication to obtain correct genome termini for example. Such blunt end dsRNA (i.e., exogenous viral dsRNA) could be recognized by the helicase domain of arthropod Dcr2. In vertebrate cells, dsRNA sensors RIG-I and MDA-5 were found to be involved in activating the type I interferon response in response to SFV [58]. Common aspects between RIG-I and arthropod Dcr2 dsRNA recognition have been suggested [55,59]. Our analysis of vsiRNA distribution, overlap, and phasing during SFV infection would suggest that processive viral dsRNA cleavage underpins the bulk of true 21 nt vsiRNA production in this *Ae*. *aegypti* cell system. The Platform-PAZ domain should be explored further in Aaeg Dcr2 relative to our findings to assess its potential contribution in distributive dsRNA targeting and cleavage [38,40,60]. Although SFV is targeted in vertebrate cells by RIG-I and MDA-5, host cell RNA derived 5’-ppp dsRNA produced by the viral replicase which is mainly sensed by RIG-I, also contributes to type I interferon induction [58]. Thus, presently we can only speculate on the nature of viral dsRNA termini that may be recognised by Aaeg Dcr2. Indeed, Dmel Dcr2 has been shown to process different viral RNAs [23]. This will require further analysis.

We suggest that during SFV infection, pools of true 21 nt vsiRNAs are generated by processive cleavage, with 5–7 nt or 15–18 nt overlaps between vsiRNA sequences in a diverse pool of these vsiRNAs, and 19 nt overlaps likely representing true 21 nt vsiRNA original duplexes. Although differences between Aaeg and Dmel Dcr2, and perhaps also other unknown nuances across arthropods [57] may exist, we do not believe it likely that 5–7 nt or 15–18 nt overlaps indicate Aaeg Dcr2 cleavage products but reflect sequence overlaps in the diverse true 21 nt vsiRNA pool. Overlapping pairs analysis with Nora virus in the *D*. *melanogaster* model showed a preference for 19 nt overlap between 21 nt vsiRNAs [51], suggesting the detection of a canonical vsiRNA duplex with 2 nt overhangs, a feature also recognized in other studies [53,54]. Indeed 19 nt overlaps were also detected with functional Aaeg Dcr2, and turnover of passenger strands in the small RNA duplex may possibly be more critical than differences between Dcr2 with regards to sequence predominance. How broadly applicable our observations are (particularly for arboviruses of other families or orders) will require further comparative investigations of vsRNA data. As we identified similar patterns for true 21 nt vsiRNAs in a previous Dcr2 expression experiment in AF319 cells infected with SFV [46], we believe that our analysis points to a pattern in this specific virus-cell context. Importantly, our analysis did show that Aaeg Dcr2 produces true 21 nt vsiRNAs with high conservation between generated sequences for WT and mutant Y232G. The use of true 21 vsiRNAs further downstream in exo-siRNA by Ago2 should also be explored further with regards to their importance during antiviral activity. This is in line with previous work, which showed that Ago2 silencing results in increased alphavirus replication in mosquitoes and mosquito cells [46,61–67]. Those findings, with the data of this study, suggest that a functional Dcr2 and complete exo-siRNA pathway are required to control SFV replication in *Ae*. *aegypti* cells.

Intriguingly, in the presence of a dysfunctional Aaeg Dcr2, 21 nt vsRNAs showed a signature typical of vpiRNAs with a ping-pong signature motif and 10 nt overlap. We propose the name vsi-piRNAs for these atypical 21 nt vsRNAs, as opposed to true Dcr2-produced true 21 nt vsiRNAs. A possible reason for this may be enhanced viral replication due to a dysfunctional Dcr2 triggering the piRNA pathway to overcompensate as the “next” immune response to the accumulation of viral dsRNA. The induction of this pathway is indeed shown by vpiRNAs observed in the small RNA sets of AF319 cells transiently expressing mutant Dcr2 or WT Dcr2. We cannot exclude the possibility that mutant Dcr2 could still cleave viral dsRNAs and that these fragments may feed into the piRNA pathway. Studies in whitefly (*Bemisia tabaci*) have suggested such a scenario, where endogenous siRNA loci are possible sources of piRNAs [68]. It is still unclear whether crosstalk between the exo-siRNA and piRNA pathways occurs during arbovirus infection of mosquitoes. Perhaps vsi-piRNAs are generally produced in smaller quantities, and because of functional Dcr2 dsRNA cleavage, these are masked by the abundance of canonical true 21 nt vsiRNAs, as shown by overlapping pairs analysis. Moreover, it has been shown that infection of C6/36 and C7/10 *Ae*. *aelbopictus* cells (both deficient in Dcr2 activity) with CHIKV resulted in the production of 21 nt vsRNAs with the characteristic A_10_/U_1_ motif, not observed in exo-siRNA functional U4.4 cells [43]. Thus vsi-piRNAs may be common in alphavirus infection of mosquito cells. In particular, Dcr2 in C7/10 cells contains a deletion between the DUF and PAZ domains, different from the conserved residues investigated in this study. Taken together with our findings, this suggests that the overall activity of Dcr2 is not limited to its functional domains, but also depends on its inter domain integrity. Nonetheless, the relevance of these findings needs further exploration.

Production of PIWI knockout cell lines derived from AF5 cells or sequencing of Ago2-associated vsRNA may clarify the contribution of 21 nt vsi-piRNAs to antiviral responses and allow the analysis of only true 21 nt vsiRNAs. Though it cannot be excluded that such vsi-piRNAs are still loaded into Ago2. Nonetheless, it may be worth considering such approaches for future work. The absence of the +1U bias in 21 nt vsi-piRNAs, which were readily observed in SFV-infected cells with mutant Dcr2, makes it at present difficult to conclude on their origin and/or processing. They may be products of vpiRNA degradation, perhaps post-processing by the recently identified *Ae*. *aegypti* ortholog of Zucchini, and/or by the corresponding ortholog Nibbler which has 3’-5’ exonuclease activity [52]. However, this would require further studies, such as silencing the expression of these proteins followed by small RNA sequence analysis. Other processing mechanisms and/or origins cannot be excluded at present. Importantly, we observed similar aspects and features for vpiRNAs derived from SINV and SFV infected mosquito cells, suggesting that mechanisms involved in alphavirus vpiRNA production are comparable.

The tools used here will expand future studies of other arboviruses within the context of mosquito vectors and assess Dcr2 functionality and antiviral hierarchy in the exo-siRNA pathway against different viral families or orders. Alongside a recently developed and characterized Ago2 knockout cell line [67] and exo-siRNA pathway associated proteins [56,69], this toolset now allows in-depth investigations of the exo-siRNA pathway during arbovirus infection in mosquito cells. Improvements of the systems applied here for future studies could involve the production of stable lines expressing Dcr2 in AF319 cells to bypass any issues related to transfection of expression plasmids, for example. Indeed, relative FFLuc levels following SFV-FFLuc infection (at MOI = 0.01) at 48 hpi were approximately 9-fold higher in AF319 cells compared to AF5 [46]. This suggests that increased Dcr2 activity than what has been observed following transfection may be possible and desirable, or even necessary depending on the experimental conditions or set up. Nonetheless, the approach described in this study did allow to reconstitute Dcr2 activity, and studies of mutants in the context of SFV infection is clearly working. Future work will also need to assess the role of the PAZ domain to vsRNA production, which was not investigated here, although our data suggest a major contribution of the helicase domain. Beyond investigations of exo-siRNA response, the introduction of the Y232G mutation that allows some functionality into Dcr2 of live mosquitoes may be an interesting prospect for vector control. Perhaps the development of an *Ae*. *aegypti* mosquito line with a Dcr2 Y232G mutation may result in an attenuated but not fully weakened vector that may succumb more easily to arbovirus infection. This will require *in vivo* investigations in the future.

## Materials and methods

### Cells

The Dcr2 knockout cell line AF319 used in this study was previously developed; it is derived from *Ae*. *aegypti* AF5 cells (a clone derived from the Aag2 cell line; provided by Kevin Maringer, The Pirbright Institute, UK) [46]. AF319 cells were grown in Leibovitz’s L-15 medium plus GlutaMax (Gibco) with 10% tryptose phosphate broth (TPB; Gibco), 10% fetal bovine serum (Gibco), and penicillin-streptomycin (pen-strep; 100 U/mL-100 μg/mL; Gibco) at 28°C [46,70]_._ These cell lines are available as Aag2-AF319 (ECACC 19022602) and Aag2-AF5 (ECACC 19022601) through Public Health England. Baby hamster kidney cells (BHK-21; a commonly used cell line available at the MRC-University of Glasgow Centre for Virus Research, UK) were grown in Glasgow Minimum Essential Medium (GMEM; Gibco) supplemented with 10% TPB, 10% newborn calf serum (Gibco), and pen-strep at 37°C with 5% CO_2_.

### Viruses

SFV4 (abbreviated to SFV; GenBank ID: KP699763) and SFV4(3H)-*FFLuc* (abbreviated to SFV-FFLuc; reporter virus expressing firefly luciferase, FFLuc) have been previously described and were produced from icDNA in BHK-21 cells with virus plaque assays done as previously described [46,71].

### Plasmids and mutagenesis

Site-directed mutagenesis of a previously developed pPUb-myc-Dcr2 [46] expression plasmid of a myc-tagged WT Aaeg Dcr2 (GenBank ID: AAW48725; coding sequence GenBank ID: AY713296) under the control of PUb promoter [72] was performed. In-Fusion cloning (Takara Bio) was performed following the manufacturer’s suggestions using mutagenesis primers designed with 15 bp extensions homologous to target vector ends (S5 Table). The mutations were based on previous studies on Dmel Dcr2 that disrupted its activity. The helicase domain mutations (K39N, Y232G, and G448R; Fig 1B) were part of known conserved amino acid motifs I, IV, and VI [47] across helicase sequences that have been identified in Dmel Dcr2 [35,37,38,40]. The RNase IIIA and IIB mutations (D1198A, E1341A, D1444A, and E1548A; Fig 1B) were based on conserved residues observed during domain IIIA and IIIB dimerization that have been investigated in Dmel Dcr2 and Dcr1 [37,48].

To identify the conserved residues for mutagenesis in Aaeg Dcr2, multiple sequence alignment of reference sequences of Dmel Dcr2 (GenBank ID: NP_523778), *H*. *sapiens* Dcr1 (GenBank ID: NP_085124), and *Ae*. *albopictus* Dcr2 (GenBank ID: NP_001339903) together with Aaeg Dcr2 WT was performed (Fig 1B, top panel) using Clustal Omega in Geneious Prime v.2021.1.1. For the helicase mutants, PCR with mutagenesis primers was performed on pPUb-myc-Dcr2 and allowed to recombine after In-Fusion reaction. For the RNase III mutants, the mutations were collectively introduced using a single construct (mtR3) from a synthetic dsDNA fragment (Eurofins). The pPUb-myc-Dcr2 plasmid was first linearized by inverse PCR, and the PCR amplified mtR3 construct mixed in an In-Fusion reaction (Takara Bio) to allow recombination.

For mutations based on *Dcr2* haplotypes (S2 Table) identified from *Ae*. *aegypti* mosquitoes collected from various geographical origins, three mutations in the helicase domain were generated, N246D (negative net charge), Q253K (positive net charge), and N246D/Q253K (neutral net charge). One mutation in the RNase IIIB domain H1514N was also generated. These were based on identified SNPs (S3 Table) between the haplotype sequences and a reference WT *Dcr2* (GenBank ID: AY713296). In making the constructs, pPUb-myc-Dcr2 was first subjected to double restriction enzyme digest using NruI and Esp3I (New England Biolabs) for the helicase mutations or SmaI and FseI (New England Biolabs) for the RNase IIIB mutant. The double restriction enzyme digest linearized the plasmid and excised the fragments of *Dcr2* intended for PCR mutagenesis. The purified PCR products were then used in different combinations with the linearized backbone during In-Fusion cloning. All plasmid constructs were transformed in Stellar Competent Cells (Takara Bio) and purified using PureLink HiPure Plasmid Maxiprep kit (Thermo Fisher Scientific) following manufacturer’s protocol. All PCRs were carried out using CloneAmp HiFi PCR Premix (Takara Bio) as per manufacturer’s instructions. The mutations in all the constructs were verified through Sanger sequencing. The firefly (FFLuc; pIZ-Fluc) and *Renilla* luciferase (RLuc; pAcIE1-Rluc) expressing plasmids used for the RNAi reporter assay have been previously described [73]. A pPUb-myc-eGFP plasmid encoding an enhanced Green Fluorescent Protein (eGFP) was used as control [46].

### dsRNA production

Primers flanked with T7 RNA polymerase promoters previously described [46,69] were used to amplify FFLuc (dsFLuc) or LacZ (dsLacZ) for *in vitro* transcription using MEGAscript RNAi kit (Thermo Fisher Scientific) as per manufacturer’s guidelines. PCR products were treated with Dnase I and Rnase A followed by column purification of dsRNA.

### RNAi reporter assay

To determine the efficiency of Aaeg Dcr2 activity, an RNAi reporter assay was performed as previously described [46]. Briefly, AF319 cells at 2.5 x 10^5^ cells/well were seeded in 24-well plates and allowed to settle for 24 h. The cells were then co-transfected with 1 μg WT or mutant Dcr2 expression plasmids (with pPUb-myc-eGFP as control), 150 ng pIZ-Fluc, and 50 ng pAcIE1-Rluc plasmids together with 1 ng dsRNA targeting FFLuc (dsFLuc) or dsRNA control (dsLacZ) and 2 μL DharmaFECT 2 (Horizon Discovery). To verify activity of the RNAi pathway at the post-dsRNA cleavage step, the cells were co-transfected with the expression plasmids as above and 1 ng siRNA targeting FFLuc (siFLuc) or siRNA control (siHyg). Cells were lysed at 24 hpt with 1X Passive Lysis Buffer (PLB; Promega), and FFLuc/RLuc levels measured using a Dual-Luciferase Reporter Assay System (Promega) observing the manufacturer’s protocols in a luminometer (GloMax Multi-Detection System with Dual Injectors; Promega).

### Virus infection

As above, 2.5 x 10^5^ AF319 cells/well in a 24-well plate were seeded, and the same amount of WT or mutant Dcr2 and eGFP (control) expression plasmids were transfected 24 h later. To test antiviral function against SFV, cells were infected at 24 hpt with SFV-FFLuc (MOI = 0.1) for 48 h at 28°C. To measure FFLuc levels from SFV-FFLuc infection, the Luciferase Assay System (Promega) was used following the manufacturer’s protocol. For small RNA sequencing samples, AF319 cells as mentioned above were transfected with 1 μg WT or mutant Dcr2 plasmid and followed by infection at 48 hpt with SFV (MOI = 1) for 48 h. Seven corresponding wells per WT or mutant Dcr2-transfected cells were pooled for total RNA extraction using TRIzol (Thermo Fisher Scientific) with glycogen as carrier, according to the manufacturer’s protocol.

### Western blot analysis

Cells were lysed with 1X Bolt sample reducing agent and 1X Bolt LDS sample buffer (Invitrogen), and lysate separated on Bolt 4–12% Bis-Tris Plus gels (Invitrogen) and transferred to 0.45 μm nitrocellulose membrane (Thermo Fisher Scientific) using a Trans-Blot SD Semi-Dry Transfer Cell (BioRad). Membranes were then blocked with 5% (w/v) non-fat dry milk in Tween-PBS (PBS with 0.1% Tween 20) for at least 1 h, and proteins of interest probed with mouse anti-myc tag antibody (1:2000; Abcam) and rabbit anti-γ tubulin antibody (1:2000; Sigma-Aldrich) in 2% (w/v) non-fat dry milk in Tween-PBS for 1 h. Three 10 minute Tween-PBS washes were carried out before incubation with goat anti-mouse DyLight 800 (1:5000; Invitrogen) and goat anti-rabbit DyLight 680 (1:5000; Invitrogen) secondary antibody conjugated with a near fluorescent dye in 2% (w/v) non-fat dry milk in Tween-PBS for 1 h. Membranes were again washed three times with Tween-PBS and viewed using Odyssey CLx with images analyzed using Image Studio Lite v.5.2.5 (LI-COR Biosciences).

### Sequencing of *Dcr2* and analysis

Natural genetic polymorphisms in Aaeg *Dcr2* gene were surveyed in early-generation colonies (<10 laboratory generations) of mosquitoes collected from Cameroon (Ben; Bénoué National Park, 2014), Gabon (Lope; Lopé National Park, 2014), Cambodia (Rat; Ratanakiri province, 2015), Cambodia (KC; Kampong Cham province, 2014), Guadeloupe (Guad; Saint François, 2015) and French Guiana (Cay; Cayenne, 2015). Before *Dcr2* sequencing, mosquito colonies were reared under controlled insectary conditions (28°C, 12 h:12 h light: dark cycle, and 70% relative humidity). Total RNA was purified from 10–15 adult females per colony using NucleoSpin RNA kit (Macherey-Nagel) and reverse transcribed into cDNA with oligo(dT) using M-MLV reverse transcriptase (Invitrogen) following manufacturer’s instructions. Full-length *Dcr2* cDNA was amplified by PCR using Q5 High Fidelity DNA polymerase (New England Biolabs) and custom forward and reverse primers (S5 Table). PCR products were purified with ChargeSwitch PCR Clean-Up Kit (Invitrogen), and the final concentration adjusted to 2 ng/μL following Qubit fluorometric quantification (Invitrogen). Multiplexed libraries were prepared using Nextera XT DNA Library Preparation Kit (Illumina) according to manufacturer’s instructions and sequenced on an Illumina NextSeq 500 (2x75 bp cycles, paired ends). Raw reads were demultiplexed using bcl2fastq v.2.15.0 (Illumina) and trimmed to remove Illumina adaptor sequences using Trimmomatic v.0.33 [74]. Clean reads were aligned to the VectorBase reference *Dcr2* sequence, AAEL006794, to extract unphased genotypes for each sample. Phased haplotypes were reconstructed using the PHASE algorithm implemented in DnaSP v.5 [75]. Thirteen *Dcr2* haplotype sequences were identified (GenBank ID: MW924847 to MW924859; S2 Table). To determine SNPS (Fig 2A and S3 Table), the 13 haplotype sequences were aligned to a WT *Dcr2* sequence (GenBank ID: AY713296, previously cloned into by pPUb-myc-Dcr2) using MUSCLE [76]. Putative amino acid sequences of haplotypes were used to generate unrooted phylogenetic trees constructed using the Neighbor-Joining method following the Jukes-Cantor model with 1000 bootstrap replicates [76–78]. The consensus tree (Fig 2B) is presented with corresponding % consensus support for each node and substitution rates. All sequences were analyzed and visualized in Geneious Prime (v.2020.1). *In silico* analysis prediction on the effect of the different mutations on Aaeg Dcr2 function (S1 Table) was performed in SIFT [49] available at https://sift.bii.a-star.edu.sg/.

### Generating a putative Aaeg Dcr2 structure

The WT sequence of Dcr2 (GenBank ID: GenBank record AAW48725) was issued as a query to DeepMind’s AlphaFold structure inference algorithm v.2.0.1 [79], using template models 1 through 5 and not restricting available templates. The resulting models were examined and in accordance with overall model quality predictions summarised in mean pLDDT value with the highest quality model selected for further analysis. Structural analyses were performed in ChimeraX [80]. Domain positions were assigned and labeled by color based on GenBank sequence annotations and predictions from InterProScan in Geneious Prime v.2021.1.1. Residues of interest were also labeled and contacts, hydrogen bonds, and distances estimated using built-in functions. Plots of error metrics (predicted local distance difference test and predicted aligned error) were produced using AlphaPickle [81].

### Small RNA sequencing and analysis

RNA library preparations and sequencing reactions were carried out by GENEWIZ, LLC (South Plainfield, NJ, USA). Initial RNA and final DNA samples were quantified using Qubit 2.0 Fluorometer (Life Technologies), and RNA integrity was checked with 4200 TapeStation (Agilent Technologies). The small RNA sequencing library was prepared using Illumina TruSeq Small RNA library Prep Kit as per the manufacturer’s specifications. The final DNA library was also validated by qPCR (KAPA Biosystems). Sequencing libraries were multiplexed and clustered to a flowcell. After clustering, the flowcell was loaded to an Illumina HiSeq 4000 according to the manufacturer’s instructions. The samples were sequenced using a 2x150bp paired-end configuration, with data trimmed to 2x50bp paired-end reads. The HiSeq Control Software conducted image analysis and base calling. The generated raw sequence data (.bcl files) from Illumina HiSeq was converted to fastq files and de-multiplexed using Illumina bcl2fastq 2.17 software with one mismatch allowed for index sequence identification. The Illumina TruSeq small RNA 3’ adapter (5’-TGGAATTCTCGGGTGCCAAGG-3’) was trimmed from fastq basecalled libraries using the fastx toolkit (Galaxy Version 1.0.3; retrieved from http://hannonlab.cshl.edu/fastx_toolkit/). Only reads containing the adapters that were 16 nt in length or greater and without ambiguous nucleotides were kept for downstream analysis. Clean reads were then mapped against the SFV genome (GenBank ID: KP699763) using Bowtie2 (Galaxy Version 2.3.4.3) [82], with the sensitive mapping flag (—sensitive). Aligned reads outputted using the–al flag for downstream analysis. The trimming and mapping statistics summary is available in S4 Table. Only forward reads were used for the analysis, and data were deposited in the NCBI Biosample database (BioProject ID: PRJNA705585).

For the analysis of host RNAi responses to SFV, individual binary alignment files for small RNA sizes for SFV derived vsiRNA (21 nt), and vpiRNA (24–30 nt) were made using BAM filter (Galaxy version 0.5.9) from Bowtie2 output BAM files. For visualization of vsiRNA and vpiRNA mapping profiles, coverage statistics for each position of the SFV genome were extracted using bedtools (v.2.27.1) genome coverage tool [83] and visualized using GraphPad Prism (v.7). To obtain nucleotide position biases in vsi/vpiRNAs, extracted positive-sense and negative-sense mapped reads were converted to fasta and visualized using WebLogo3 [84]. For determination of overlapping vsi/vpiRNAs, overlapping pairs and overlap probabilities were determined from Bowtie2 BAM files using the small RNA signatures tool (v.3.2.1) hosted on the Mississippi Galaxy server (https://mississippi.sorbonne-universite.fr/) [51].

For the analysis of downstream SFV genomic nucleotide bias (+1–4) of SFV small RNA reads, 21 nt, representing true vsiRNAs or vsi-piRNAs, and 25–30 nt, representing vpiRNAs, were extracted from each genome orientation of BAM files and converted to fasta format. The downstream +1–4 nucleotides in the SFV genome of exact matches were iteratively extracted using grep (v2.20). Biases in resultant downstream +1–4 nucleotides were visualised using WebLogo3 [84].

To examine the broad diversity of SFV-derived small RNA species, we treated all unique 16–46 nt mapped reads as discrete vsRNA and produced counts tables for all extracted SFV small RNAs. Mapped fastq reads from individual libraries were converted, processed, and enumerated in bash (v.3.2). The resulting dimensions of the counts table for all 16–46 nt vsRNA species and 21 nt vsRNAs were 398,279 and 23,934, respectively. Count tables were then imported to the differential gene expression package edgeR (v.3.30.3) [85] in the R Studio (v.1.3.1073) environment and normalized for library size using the weighted TMM method in calcNormFactors() and filtered for a minimum count of 5 with filterByExpr(). To explore the relationship between samples, an MDS plot of the edgeR normalized count tables with plotMDS(top = 500) of both the SFV vsRNAs and 21 nt vsRNAs was constructed. A heatmap of the log-transformed TMM normalized 21 nt vsRNAs was produced using pheatmap (v.1.0.12; https://CRAN.R-project.org/package=pheatmap). Hierarchical clustering was performed in pheatmap by calculating a distance matrix using Pearson correlation distance ("correlation"), and clustered using Ward’s minimum variance method (“ward.d2”).

### Data analyses

All experiments were performed in n = 3 independent biological repeats with 4 technical repeats except for the small RNA sequencing and immunoblot analyses which were performed with duplicate samples. Luciferase readings for RNAi reporter assay were expressed as FFLuc/RLuc ratio to account for transfection efficiency and normalized to the negative control (dsLacZ) readings set to 1, and relative light units were presented as mean±SEM. SFV-FFLuc replication measured as FFLuc readings and presented as mean±SEM light units relative to the negative control (peGFP) set to 1. Statistical significance was assessed by a two-tailed Students t-test or two-way ANOVA with Tukey’s Multiple Comparison, when appropriate at alpha = 0.05. All data analyses and visualizations were performed in GraphPad Prism (v.7).

## Supporting information

S1 Table SIFT scores of Dcr2 loss-of-function mutations and natural haplotype variants. (DOCX)

S2 Table *Dcr2* haplotype sequences from field-derived *Ae*. *aegypti* mosquitoes. (DOCX)

S3 Table SNPs identified from *Dcr2* haplotypes. Substitutions are relative to a reference WT *Dcr2* sequence (GenBank ID: AY713296).
(DOCX)

S4 Table Small RNA library metadata and mapping analysis. Numbers for each independent replicate are shown.
(DOCX)

S5 Table Primers used in this study. Mutations introduced are in red, homologous overlaps are in bold. (DOCX)

S1 Fig Loss-of-function mutations in both helicase and RNase III domains do not affect exo-siRNA pathway induction by siRNAs. siRNA silencing activity of mutant Dcr2 determined by RNAi reporter assay using AF319 cells co-transfected with mutant or WT Dcr2 plasmid (peGFP as control) and FFLuc and RLuc reporter plasmids with siRNA targeting FFLuc (siFLuc) or siHyg (non-targeting control). At 24 hpt, luciferase levels measured and presented as mean±SEM relative light units (FFLuc/RLuc) versus siHyg set to 1 from n = 3 independent repeats with ** = p<0.01 versus controls according to two-way ANOVA.
(TIF)

S2 Fig *Dcr2* haplotypes from field-derived mosquitoes. Pairwise comparisons (% identity) of *Dcr2* haplotypes identified from different mosquito populations including a reference WT *Dcr2* (AY713296).
(TIF)

S3 Fig Confidence metrics and quality assurance of AlphaFold structure predictions for Aaeg Dcr2. (A) Predicted local distance difference test (pLDDT) values for AlphaFold model of WT Aaeg Dcr2 (GenBank ID: AAW48725) displaying the local confidence of the predicted conformation with respect to neighboring residues plotted against the residue index and (B) highlighted on the structure of the molecule with confidence values mapped to regions of interest. (C) AlphaFold model of Aaeg Dcr2 fitted in electron density map (in grey) of Dmel Dcr2 calculated by Sinha et al. [38] (EMDB entry 7291, contour level 0.0066). “Body” module (residues 650–1025 and 1150–1658) cyan ribbon; “foot” module (residues 1–550) pink ribbon; flexible linker (residues 1025–1150) grey ribbon; DUF (residues 550–650) green ribbon and star. (D) Predicted alignment error (PAE) plot, showing the predicted deviation from modelled positions for each residue pair in the AlphaFold model of Aaeg Dcr2 in Å. Regions of higher confidence corresponding to “body” and “foot” modules and DUF are highlighted in cyan, pink and green, respectively.
(TIF)

S4 Fig SFV-derived vpiRNAs detected from small RNA sequencing of Dcr2 samples. (A) Distribution of SFV-derived piRNAs (24–30 nt) mapped to the genome (magenta) and anti-genome (cyan) presented as mean number of mapped reads from n = 2 independent repeats. The SFV genome organization (top panel) is shown for reference. (B) Sequence logos of vpiRNAs (27 nt) mapping to the genome (left panel) or anti-genome (right panel) showing the signature A_10_/U_1_ motif. Replicates (n = 2) are shown with the overall height of the stack, indicating conservation presented as bits and height symbols reflecting the relative frequency of the corresponding nucleotide at a given nucleotide position.
(TIF)

S5 Fig Ping-pong signatures of vsi-piRNAs. Sequence logos of replicate 2 of 21 nt vsRNAs mapping to the genome (left panel) or anti-genome (right panel) of SFV from AF319 transiently expressing mutant or WT Dcr2. Overall height of the stack indicates conservation presented as bits, with the height of each symbol reflecting the relative frequency of the corresponding nucleotide at a given position. Error bars indicate an approximate Bayesian 95% credible interval.
(TIF)

S6 Fig SFV vpiRNAs (25–30 nt) have a bias towards U in the +1 position. Overall height of the stack indicates the probability of the corresponding nucleotide at a given position for both (A) genome and (B) anti-genome SFV reads. The U bias at the +1 position was not observed in 21 nt length vsRNAs from all mutant and WT Aaeg Dcr2. Reads of n = 2 replicates were pooled with the total mapped read indicated at the upper left of each graph.
(TIF)

S7 Fig Cleavage patterns of viral dsRNA during SFV infection. (A) Z-score of 21 nt vsRNAs from AF319 transiently expressing mutant or WT Dcr2, infected with SFV. Mean of n = 2 independent repeats is presented as a blue line with grey regions marking the range. (B) Re-analysis of previous data of small RNAs [46] from SFV infected AF319 (Dcr2 knockout), AF319+WT Dcr2 (Dcr2 phenotype rescue), and AF5 (parental clone of AF319) cells. Heat maps show mean overlap probabilities of z-scores of 18–30 nt SFV-derived vsRNAs with nucleotide lengths shown horizontally, and the number of nucleotide overlaps listed vertically. Red arrow labelled Dcr2 indicates the expected 2 nt overlap from dsRNA cleavage with cells boxed in black. Black arrow labelled pp shows expected 10 nt overlap from ping-pong amplification. (C) Parsed 21 nt vsRNAs from the previous analysis (in B) showing probability z-score of overlapping pairs across the number of overlapping nucleotides. (D) Normalized SFV-derived vsRNA reads (16–46 nt; left panel) and parsed 21 nt vsRNAs (right panel) expressed as Log2 counts per million.
(TIF)

S1 Movie AlphaFold predicted structure of WT Aaeg Dcr2. As determined previously (Fig 3), with positions of conserved amino acids critical for Dcr2 function and natural haplotype variants indicated.
(MP4)
