# Supplementary material for: Mutational analysis of Aedes aegypti Dicer 2 provides insights into the biogenesis of antiviral exogenous small interfering RNAs
Source: PLoS Pathog. 2022 Jan 6;18(1):e1010202. doi: 10.1371/journal.ppat.1010202 (PMC8769306; doi:10.1371/journal.ppat.1010202)
Supplement: S1 Table — (DOCX) [file ppat.1010202.s001.docx]

**S1 Table. SIFT scores of Dcr2 loss-of-function mutations and natural haplotype variants.**

| **Mutation** | **SIFT Score** | **Prediction** |
| --- | --- | --- |
| **Loss-of-function mutants** | | |
| K39N | 0.00 | Affects protein function |
| Y232G | 0.00 | Affects protein function |
| G488R | 0.00 | Affects protein function |
| D1198A | 0.00 | Affects protein function |
| E1341A | 0.00 | Affects protein function |
| D1444A | 0.00 | Affects protein function |
| E1548A | 0.00 | Affects protein function |
| **Haplotype variants (count, n=13)** | | |
| N246D (5) | 0.58 | Tolerated |
| Q253K (6) | 0.88 | Tolerated |
| H1514N (4) | 0.48 | Tolerated |
