# Supplementary material for: Mutational analysis of Aedes aegypti Dicer 2 provides insights into the biogenesis of antiviral exogenous small interfering RNAs
Source: PLoS Pathog. 2022 Jan 6;18(1):e1010202. doi: 10.1371/journal.ppat.1010202 (PMC8769306; doi:10.1371/journal.ppat.1010202)
Supplement: S2 Table — (DOCX) [file ppat.1010202.s002.docx]

**S2 Table. *Dcr2* haplotype sequences from field-derived *Ae. aegypti* mosquitoes.**

| **No.** | **Name** | **Origin** | **Year collected** | **GenBank ID** |
| --- | --- | --- | --- | --- |
| 1 | Ben_Hap3 | Bénoué National Park, Cameroon | 2014 | MW924847 |
| 2 | Ben_Hap11 | Bénoué National Park, Cameroon | 2014 | MW924848 |
| 3 | Cay_Hap1 | Cayenne, French Guiana | 2015 | MW924849 |
| 4 | Cay_Hap3 | Cayenne, French Guiana | 2015 | MW924850 |
| 5 | Cay_Hap4 | Cayenne, French Guiana | 2015 | MW924851 |
| 6 | Guad_Hap2 | Saint François, Guadeloupe | 2015 | MW924852 |
| 7 | Guad_Hap3 | Saint François, Guadeloupe | 2015 | MW924853 |
| 8 | Guad_Hap4 | Saint François, Guadeloupe | 2015 | MW924854 |
| 9 | KC_Hap6 | Kampong Cham, Cambodia | 2015 | MW924855 |
| 10 | Lope_Hap7 | Lopé National Park, Gabon | 2014 | MW924856 |
| 11 | Lope_Hap9 | Lopé National Park, Gabon | 2014 | MW924857 |
| 12 | Rat_Hap1 | Ratanakiri, Cambodia | 2015 | MW924858 |
| 13 | Rat_Hap2 | Ratanakiri, Cambodia | 2015 | MW924859 |
