# Supplementary material for: Mutational analysis of Aedes aegypti Dicer 2 provides insights into the biogenesis of antiviral exogenous small interfering RNAs
Source: PLoS Pathog. 2022 Jan 6;18(1):e1010202. doi: 10.1371/journal.ppat.1010202 (PMC8769306; doi:10.1371/journal.ppat.1010202)
Supplement: S3 Table — Substitutions are relative to a reference WT Dcr2 sequence (GenBank ID: AY713296). (DOCX) [file ppat.1010202.s003.docx]

**S3 Table. SNPs identified from *Dcr2* haplotypes.** Substitutions are relative to a reference WT *Dcr2* sequence (GenBank ID: AY713296).

| **Nucleotide position** | **WT** | **Variant** | **Amino acid change** | **Type*** | **Frequency**  **n=13** | **Strand-bias p-value** | |
| --- | --- | --- | --- | --- | --- | --- | --- |
|  |  |  |  |  |  | >50% | >65% |
| 498 | T | A | - | Transversion | 84.6% | 0.00 | 0.02 |
| 549 | A | G | - | Transition | 61.5% | 0.01 | 0.06 |
| 736 | A | G | N246D | Transition | 38.5% | 0.06 | 0.23 |
| 757 | C | A | Q253K | Transversion | 46.2% | 0.03 | 0.15 |
| 963 | A | G | - | Transition | 38.5% | 0.06 | 0.23 |
| 1134 | C | T | - | Transition | 76.9% | 0.00 | 0.03 |
| 1578 | T | C | - | Transition | 76.9% | 0.00 | 0.03 |
| 1623 | T | C | - | Transition | 46.2% | 0.03 | 0.15 |
| 1684 | T | C | - | Transition | 100% | 0.00 | 0.01 |
| 1776 | C | G | - | Transversion | 69.2% | 0.00 | 0.04 |
| 1915 | T | C | - | Transition | 92.3% | 0.00 | 0.01 |
| 2088 | A | G | - | Transition | 30.8% | 0.13 | 0.36 |
| 2160 | G | A | - | Transition | 53.8% | 0.02 | 0.10 |
| 2178 | C | T | - | Transition | 38.5% | 0.06 | 0.23 |
| 2227 | T | A | L743I | Transversion | 30.8% | 0.13 | 0.36 |
| 2379 | G | A | - | Transition | 38.5% | 0.06 | 0.23 |
| 2491 | A | G | K831E | Transition | 30.8% | 0.13 | 0.36 |
| 2724 | G | T | - | Transversion | 38.5% | 0.06 | 0.23 |
| 2895 | C | T | - | Transition | 69.2% | 0.00 | 0.04 |
| 2898 | T | A | - | Transversion | 69.2% | 0.00 | 0.04 |
| 2955 | C | T | - | Transition | 69.2% | 0.00 | 0.04 |
| 3054 | A | G | - | Transition | 38.5% | 0.06 | 0.23 |
| 3368-69 | CA | AT | T1123N | Substitution | 38.5% | 0.06 | 0.23 |
| 3421 | C | G | P1141A | Transversion | 38.5% | 0.06 | 0.23 |
| 3433 | C | T | - | Transition | 38.5% | 0.06 | 0.23 |
| 3771 | T | C | - | Transition | 84.6% | 0.00 | 0.02 |
| 3804 | T | C | - | Transition | 69.2% | 0.00 | 0.04 |
| 3897 | C | A | - | Transversion | 61.5% | 0.01 | 0.06 |
| 3942 | T | C | - | Transition | 61.5% | 0.01 | 0.06 |
| 4089 | T | C | - | Transition | 61.5% | 0.01 | 0.06 |
| 4119 | A | G | - | Transition | 30.8% | 0.13 | 0.36 |
| 4121-22 | GA | AG | R1374K | Substitution | 61.5% | 0.01 | 0.06 |
| 4216 | G | A | G1406S | Transition | 61.5% | 0.01 | 0.06 |
| 4365 | C | T | - | Transition | 46.2% | 0.03 | 0.15 |
| 4540 | C | A | H1514N | Transversion | 30.8% | 0.13 | 0.36 |
| 4653 | G | A | - | Transition | 38.5% | 0.06 | 0.23 |
| *transition/transversion ratio, R:  $\text{R}=\frac{\text{number of transitions}}{\text{number of transversions}}=\text{2.5}$ | | | | | | | |
