# Supplementary material for: Mutational analysis of Aedes aegypti Dicer 2 provides insights into the biogenesis of antiviral exogenous small interfering RNAs
Source: PLoS Pathog. 2022 Jan 6;18(1):e1010202. doi: 10.1371/journal.ppat.1010202 (PMC8769306; doi:10.1371/journal.ppat.1010202)
Supplement: S4 Table — Numbers for each independent replicate are shown. (DOCX) [file ppat.1010202.s004.docx]

**S4 Table. Small RNA library metadata and mapping analysis.** Numbers for each independent replicate are shown.

| **Sample Name** | **SRA Accession** | **Description** | **Number of reads** | **After trimming** | **Mapped to SFV (%)** |
| --- | --- | --- | --- | --- | --- |
| WTSFV1 | SRR13810522 | WT Dcr2, SFV, replicate 1 | 11,007,961 | 9,815,734 | 800,522 (8.16%) |
| WTSFV2 | SRR13810521 | WT Dcr2, SFV, replicate 2 | 10,075,210 | 9,023,167 | 993,548 (11.01%) |
| YGSFV1 | SRR13810518 | Dcr2 Y232G, SFV, replicate 1 | 10,596,091 | 9,369,052 | 1,074,814 (11.77%) |
| YGSFV2 | SRR13810517 | Dcr2 Y232G, SFV, replicate 2 | 10,242,938 | 9,132,363 | 1,166,177 (12.45%) |
| GRSFV1 | SRR13810514 | Dcr2 G488R, SFV, replicate 1 | 10,252,732 | 8,921,772 | 1,515,213 (16.98%) |
| GRSFV2 | SRR13810513 | Dcr2 G488R, SFV, replicate 2 | 11,413,786 | 10,195,070 | 1,389,860 (13.63%) |
| KNSFV1 | SRR13810520 | Dcr2 K39N, SFV, replicate 1 | 11,282,448 | 9,849,105 | 1,577,024 (16.01%) |
| KNSFV2 | SRR13810519 | Dcr2 K39N, SFV, replicate 2 | 11,428,853 | 10,082,320 | 1,475,079 (14.63%) |
| R3SFV1 | SRR13810516 | Dcr2 mtR3, SFV, replicate 1 | 9,748,576 | 8,462,149 | 1,428,761 (16.88%) |
| R3SFV2 | SRR13810515 | Dcr2 mtR3, SFV, replicate 2 | 11,007,918 | 9,770,059 | 1,218,530 (12.47%) |
