# Supplementary material for: Mutational analysis of Aedes aegypti Dicer 2 provides insights into the biogenesis of antiviral exogenous small interfering RNAs
Source: PLoS Pathog. 2022 Jan 6;18(1):e1010202. doi: 10.1371/journal.ppat.1010202 (PMC8769306; doi:10.1371/journal.ppat.1010202)
Supplement: S5 Table — (DOCX) [file ppat.1010202.s005.docx]

**S5 Table. Primers used in this study.** Mutations introduced are in red, homologous overlaps are in bold

| **Primer** | **Sequence (5’-3’)** | **Use** |
| --- | --- | --- |
| K39N_F | **GCCGGAAACACCCAT**ATCGCCCTGATGG | K39N mutation |
| K39F_R | **ATGGGTGTTTCCGGC**TCCGGTCGGC |  |
| Y232G_F | **GGTGTCTGGATCAAC**CCTTCGACTGTCACCTG | Y232G mutation |
| Y232G_R | **GTTGATCCAGACACC**AAAAGTTCATTGGGATC |  |
| G488R_F | **GTCAAAACGGAGAGC**CCGTATGAAGAACAGTCAG | G448R mutation |
| G488R_R | **GCTCTCCGTTTTGAC**TGCTGATACGACGC |  |
| mtR3_F1 | **TTTGGACTCTGGAAA**CGACTTTGC | mtR3 inverse PCR |
| mtR3_R1 | **CCAGGTCAAAAACAT**CGGACG |  |
| mtR3_F2 | **ATGTTTTTGACCTGG**AACGCTACGAATTGTTGGGCG | mtR3 insert PCR |
| mtR3_R2 | **TTTCCAGAGTCCAAA**AAGACCGCAGCCACCAGAC |  |
| N246D_R | **CTGAAAGCGTTGATG**TTATCCACGATGTCAGCCATCACAGG | N246D mutation |
| Q253Q_F | **CATCAACGCTTTCAG**TCAAACGATTGAAGAATTTCACCTTC | WT |
| N246N_R | **CTGAAAGCGTTGATG**TTGTTCACGATGTCAGCCATCACAGG | WT |
| Q253K_F | **CATCAACGCTTTCAG**TAAGACGATTGAAGAATTTCACCTTC | Q253K mutation |
| NruI_F | GGCTGGAAGAGTTCGCGAAGTACCAGGTCATTGTTTG | PCR region of interest |
| Esp3I_R | CTTCCGTCTCAGCGAAGTAAATTTTCAGTAAATGGTAC |  |
| H1514N_F | **TTCCAAGAACAAAAC**AAACACGAAATTACCGATCAG | H1514N mutation |
| H1514N_R | **GTTTTGTTCTTGGAA**CAACAC | H1514N mutation |
| SmaI_F | CCTACCATGAGTCCCGGGCAGTTAACAGATCTGCGC | PCR region of interest |
| FseI_Rev | GTCTGCTCGAAGCGGCCGGCCTTACTTGTACCTTACTTAGC |  |
| Dcr2_4 | TAATCGTGSTAKGCCGTACC | *dcr2* sequencing |
| Dcr2_5 | ATCCAACTGAACCATCATGG |  |
| Dcr2_R | TGATCTTCTCCAAACCCGCC |  |
