# Supplementary figures and images for: Mutational analysis of Aedes aegypti Dicer 2 provides insights into the biogenesis of antiviral exogenous small interfering RNAs
Source: PLoS Pathog. 2022 Jan 6;18(1):e1010202. doi: 10.1371/journal.ppat.1010202 (PMC8769306; doi:10.1371/journal.ppat.1010202)

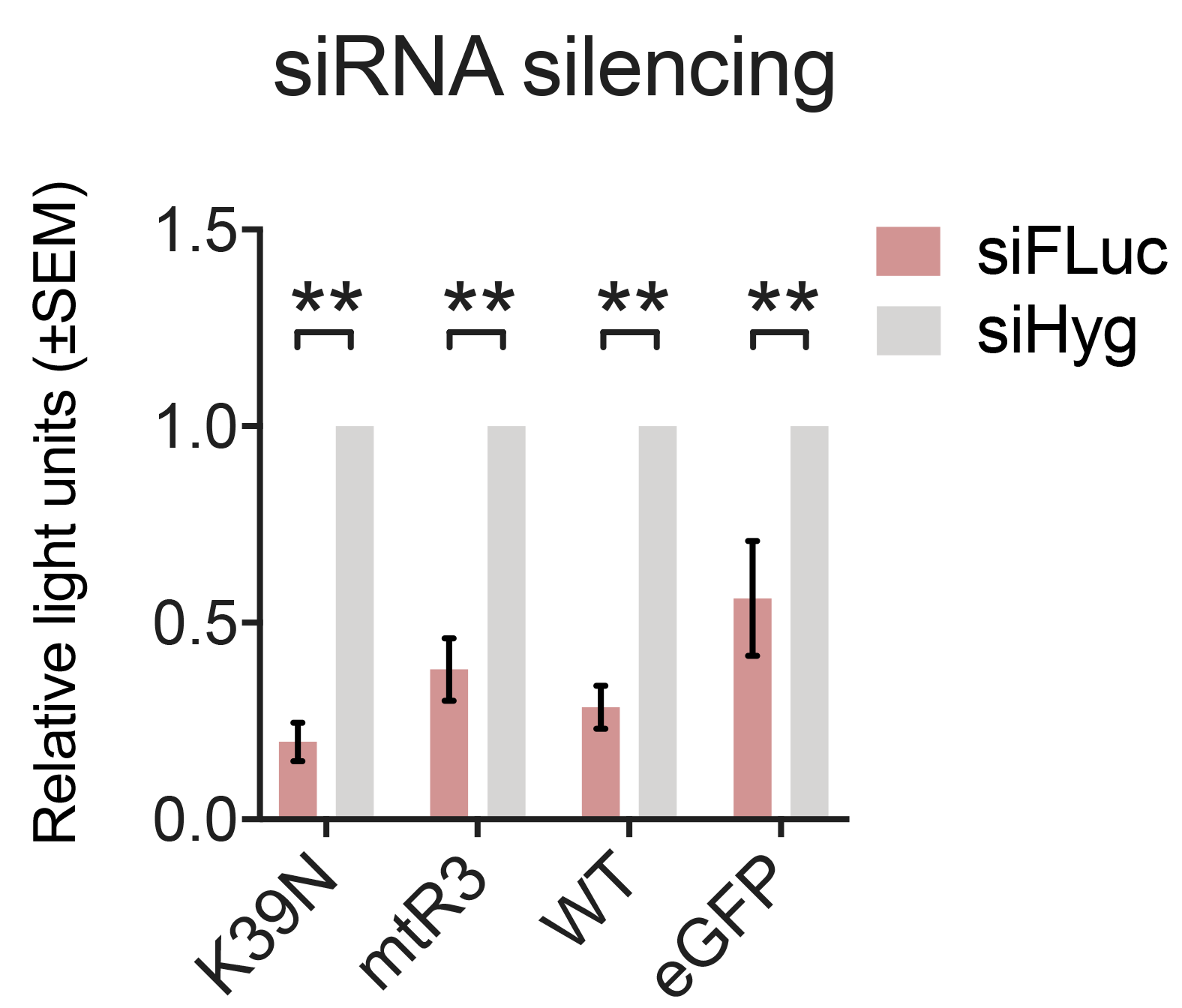

Supplement: S1 Fig — siRNA silencing activity of mutant Dcr2 determined by RNAi reporter assay using AF319 cells co-transfected with mutant or WT Dcr2 plasmid (peGFP as control) and FFLuc and RLuc reporter plasmids with siRNA targeting FFLuc (siFLuc) or siHyg (non-targeting control). At 24 hpt, luciferase levels measured and presented as mean±SEM relative light units (FFLuc/RLuc) versus siHyg set to 1 from n = 3 independent repeats with ** = p<0.01 versus controls according to two-way ANOVA. (TIF) [file ppat.1010202.s006.tif]

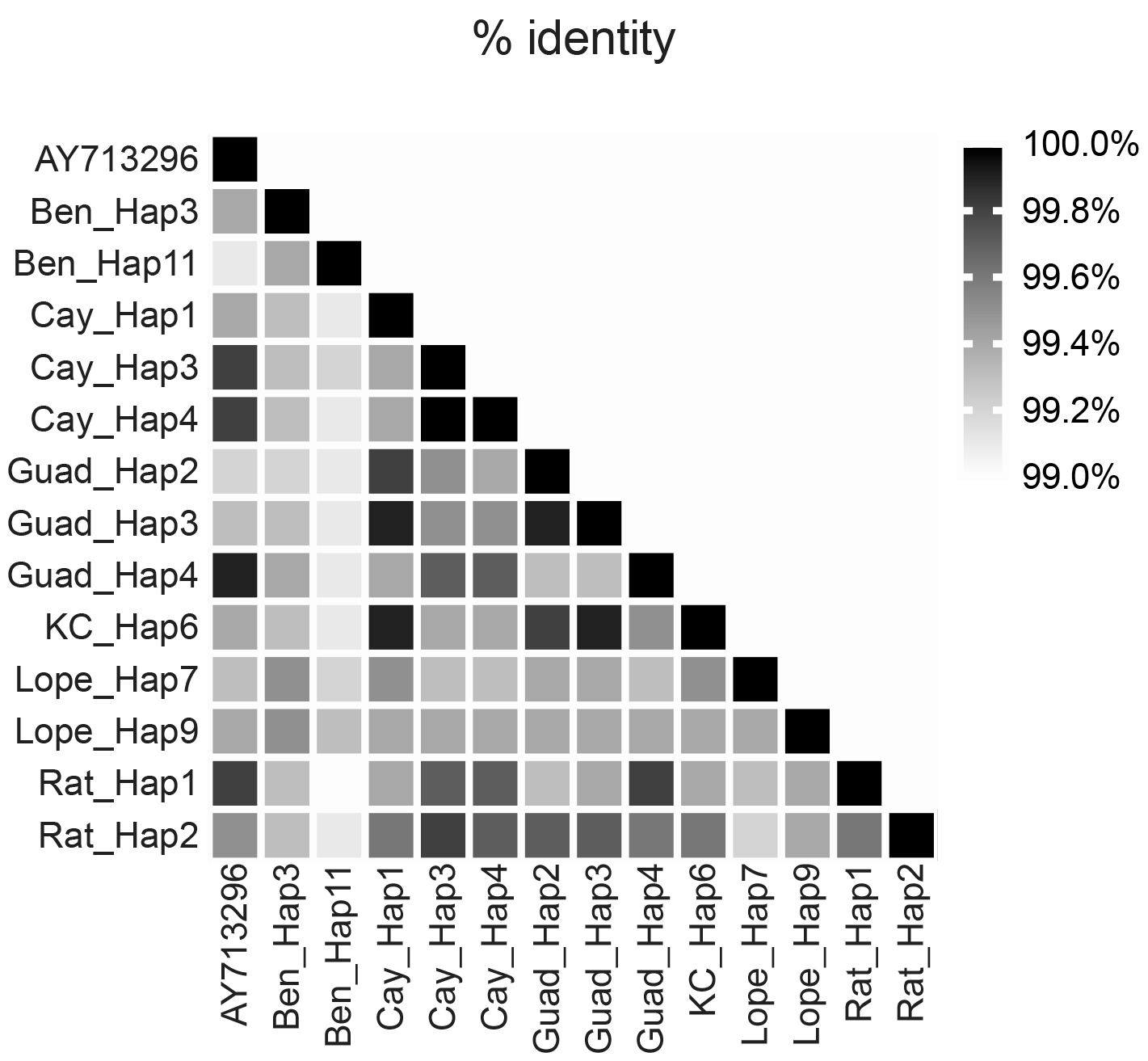

Supplement: S2 Fig — Pairwise comparisons (% identity) of Dcr2 haplotypes identified from different mosquito populations including a reference WT Dcr2 (AY713296). (TIF) [file ppat.1010202.s007.tif]

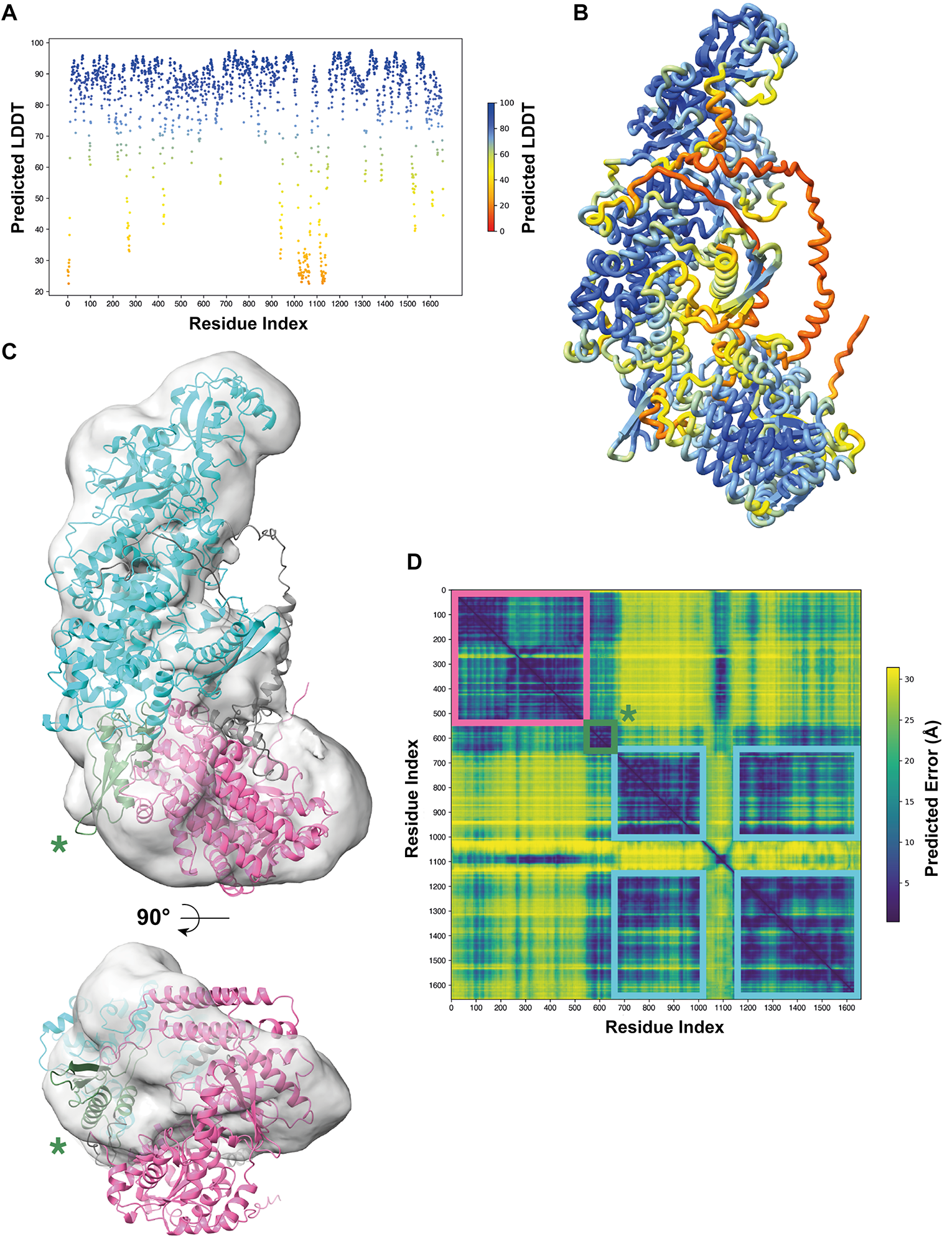

Supplement: S3 Fig — (A) Predicted local distance difference test (pLDDT) values for AlphaFold model of WT Aaeg Dcr2 (GenBank ID: AAW48725) displaying the local confidence of the predicted conformation with respect to neighboring residues plotted against the residue index and (B) highlighted on the structure of the molecule with confidence values mapped to regions of interest. (C) AlphaFold model of Aaeg Dcr2 fitted in electron density map (in grey) of Dmel Dcr2 calculated by Sinha et al. [38] (EMDB entry 7291, contour level 0.0066). “Body” module (residues 650–1025 and 1150–1658) cyan ribbon; “foot” module (residues 1–550) pink ribbon; flexible linker (residues 1025–1150) grey ribbon; DUF (residues 550–650) green ribbon and star. (D) Predicted alignment error (PAE) plot, showing the predicted deviation from modelled positions for each residue pair in the AlphaFold model of Aaeg Dcr2 in Å. Regions of higher confidence corresponding to “body” and “foot” modules and DUF are highlighted in cyan, pink and green, respectively. (TIF) [file ppat.1010202.s008.tif]

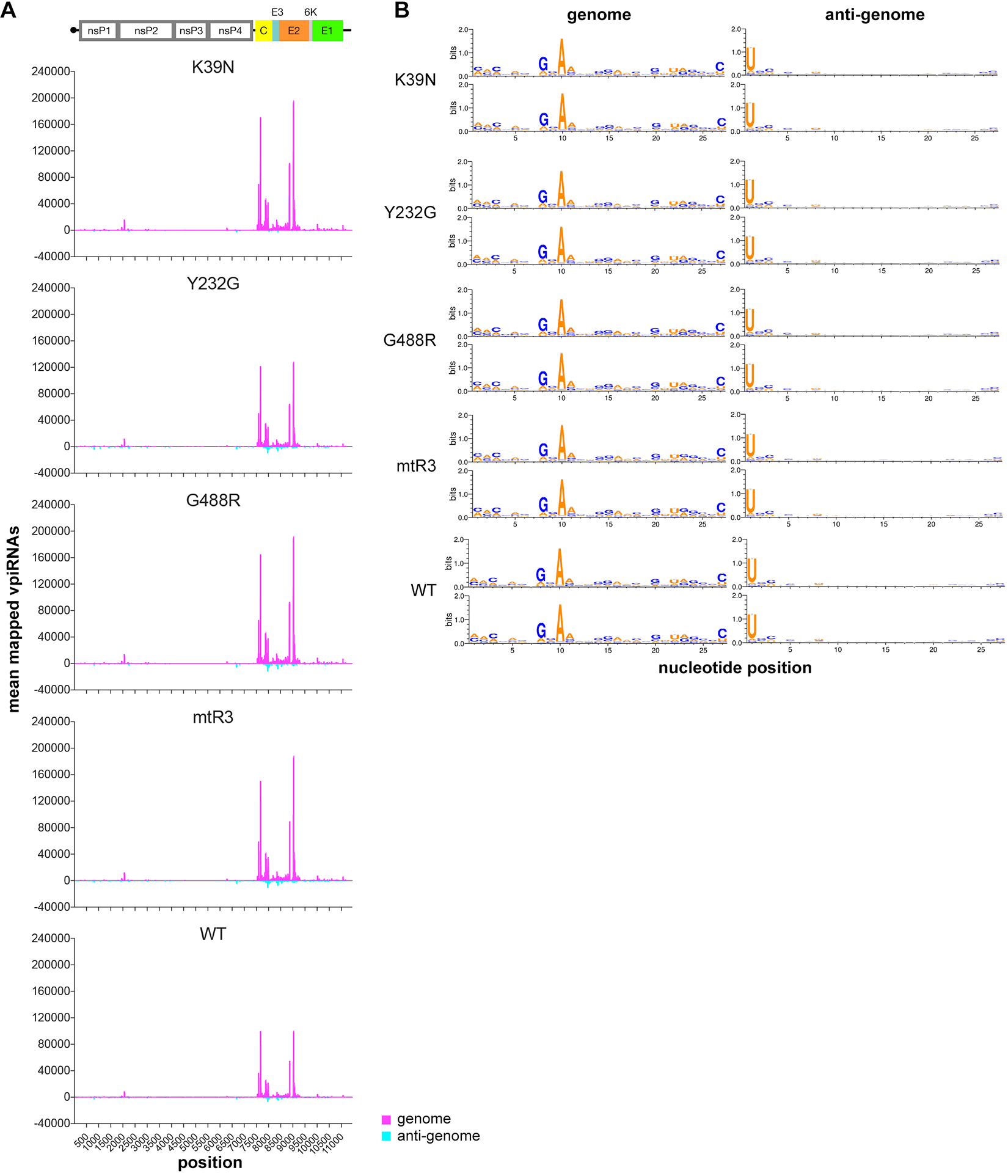

Supplement: S4 Fig — (A) Distribution of SFV-derived piRNAs (24–30 nt) mapped to the genome (magenta) and anti-genome (cyan) presented as mean number of mapped reads from n = 2 independent repeats. The SFV genome organization (top panel) is shown for reference. (B) Sequence logos of vpiRNAs (27 nt) mapping to the genome (left panel) or anti-genome (right panel) showing the signature A10/U1 motif. Replicates (n = 2) are shown with the overall height of the stack, indicating conservation presented as bits and height symbols reflecting the relative frequency of the corresponding nucleotide at a given nucleotide position. (TIF) [file ppat.1010202.s009.tif]

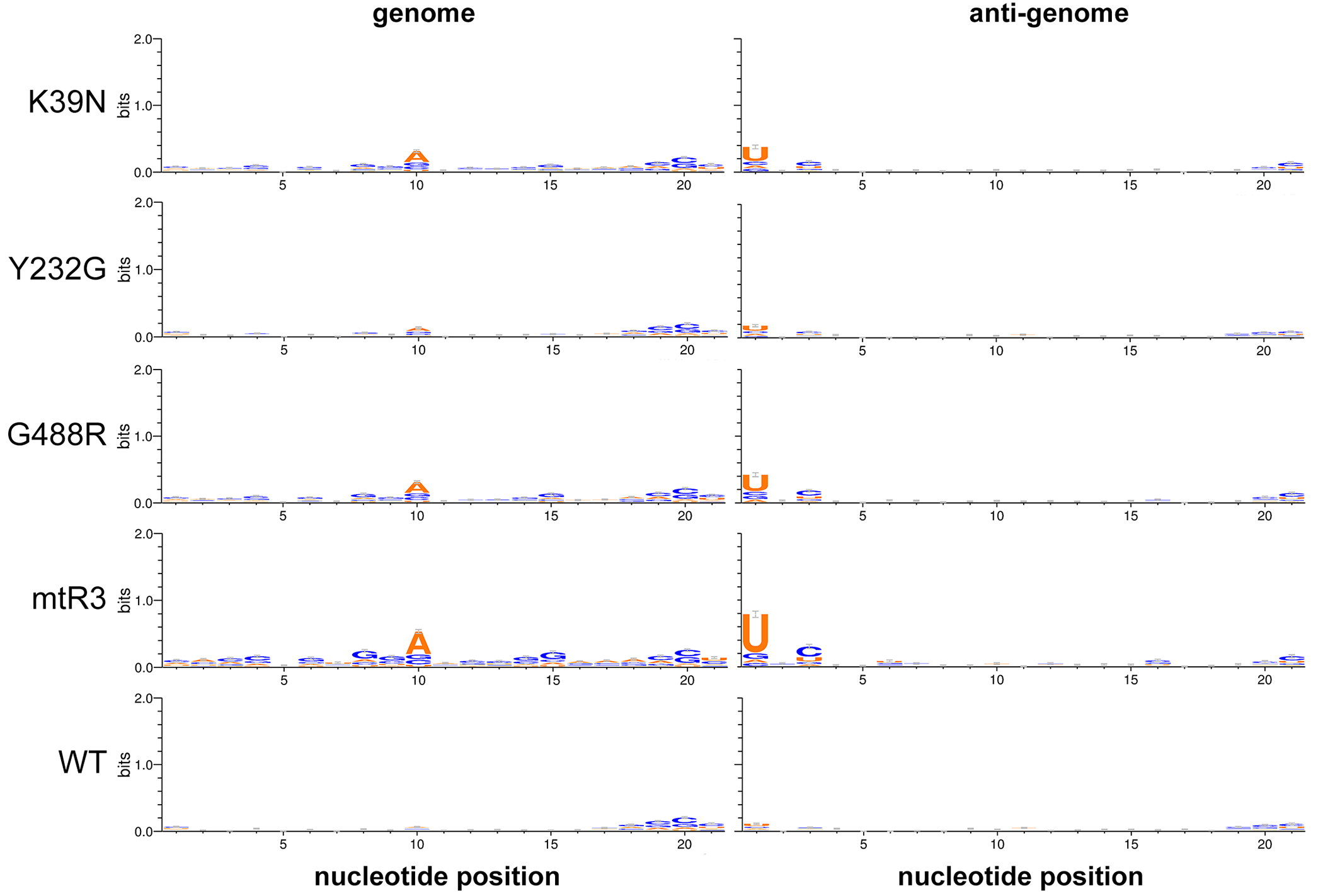

Supplement: S5 Fig — Sequence logos of replicate 2 of 21 nt vsRNAs mapping to the genome (left panel) or anti-genome (right panel) of SFV from AF319 transiently expressing mutant or WT Dcr2. Overall height of the stack indicates conservation presented as bits, with the height of each symbol reflecting the relative frequency of the corresponding nucleotide at a given position. Error bars indicate an approximate Bayesian 95% credible interval. (TIF) [file ppat.1010202.s010.tif]

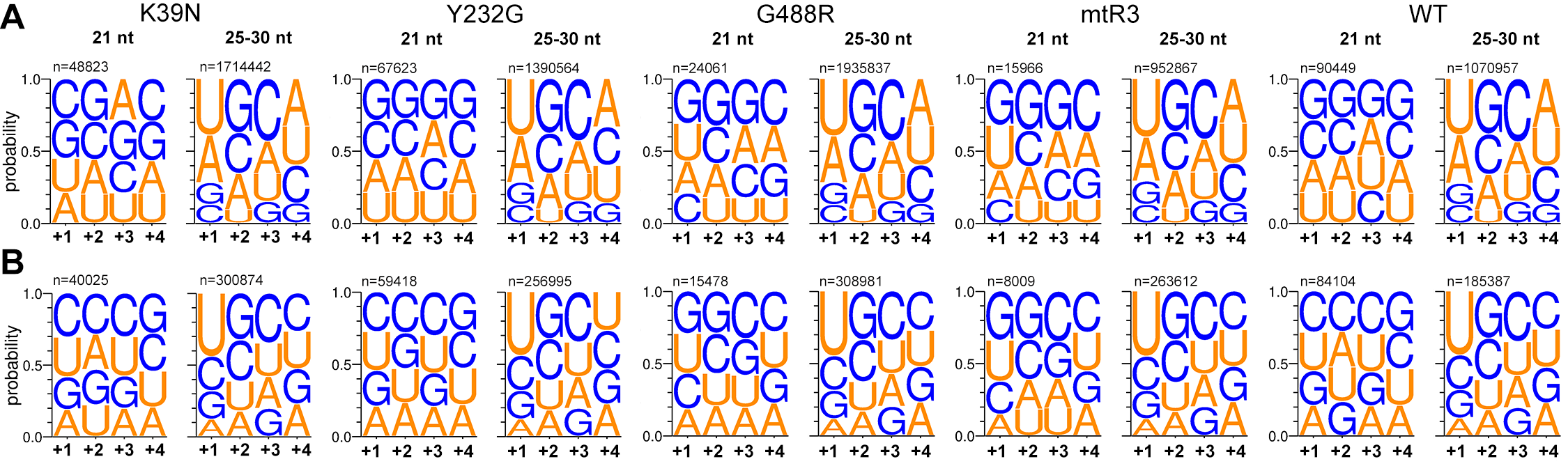

Supplement: S6 Fig — Overall height of the stack indicates the probability of the corresponding nucleotide at a given position for both (A) genome and (B) anti-genome SFV reads. The U bias at the +1 position was not observed in 21 nt length vsRNAs from all mutant and WT Aaeg Dcr2. Reads of n = 2 replicates were pooled with the total mapped read indicated at the upper left of each graph. (TIF) [file ppat.1010202.s011.tif]

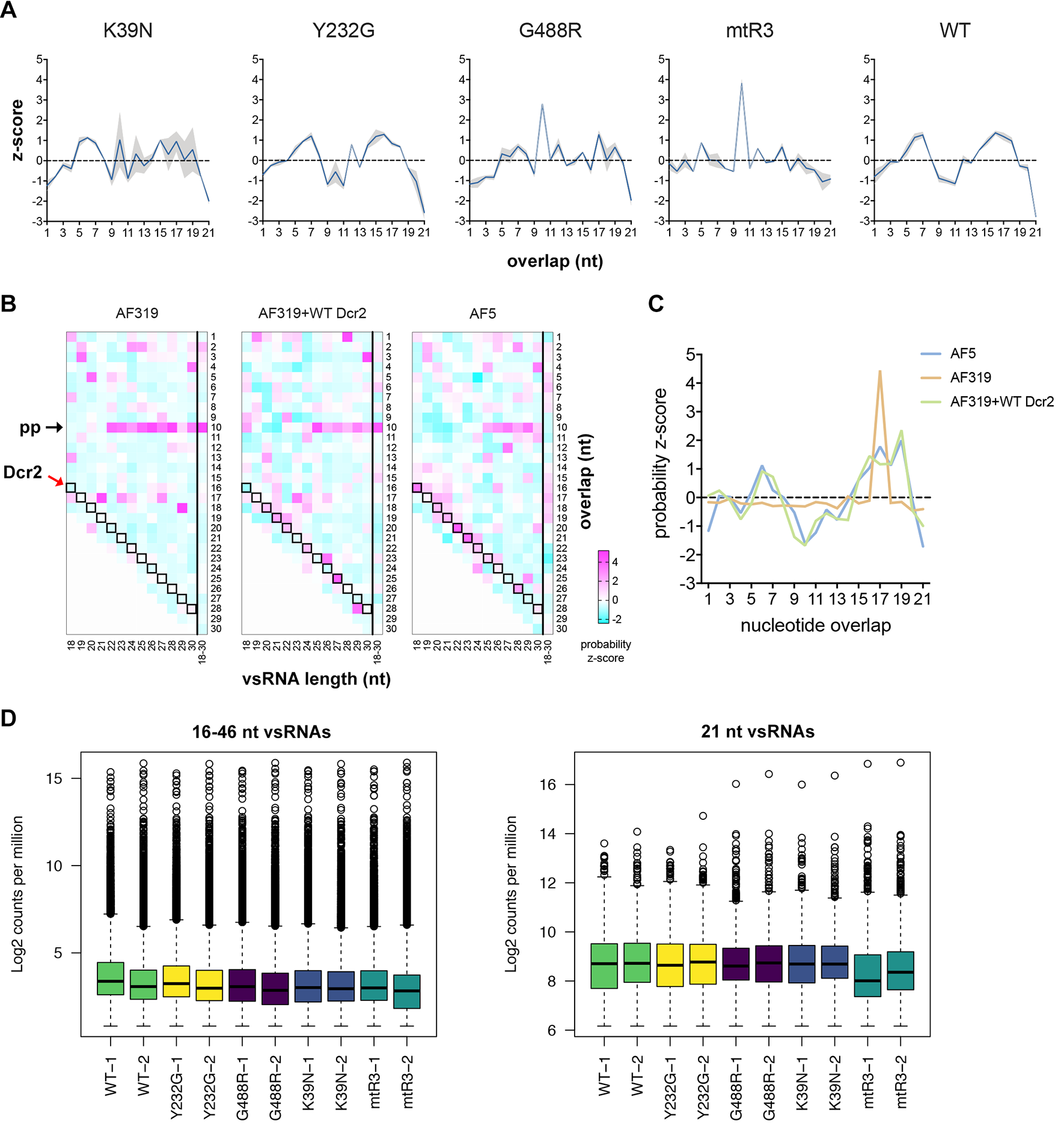

Supplement: S7 Fig — (A) Z-score of 21 nt vsRNAs from AF319 transiently expressing mutant or WT Dcr2, infected with SFV. Mean of n = 2 independent repeats is presented as a blue line with grey regions marking the range. (B) Re-analysis of previous data of small RNAs [46] from SFV infected AF319 (Dcr2 knockout), AF319+WT Dcr2 (Dcr2 phenotype rescue), and AF5 (parental clone of AF319) cells. Heat maps show mean overlap probabilities of z-scores of 18–30 nt SFV-derived vsRNAs with nucleotide lengths shown horizontally, and the number of nucleotide overlaps listed vertically. Red arrow labelled Dcr2 indicates the expected 2 nt overlap from dsRNA cleavage with cells boxed in black. Black arrow labelled pp shows expected 10 nt overlap from ping-pong amplification. (C) Parsed 21 nt vsRNAs from the previous analysis (in B) showing probability z-score of overlapping pairs across the number of overlapping nucleotides. (D) Normalized SFV-derived vsRNA reads (16–46 nt; left panel) and parsed 21 nt vsRNAs (right panel) expressed as Log2 counts per million. (TIF) [file ppat.1010202.s012.tif]
